# Astrocyte Reactivity: A Biomarker for Retinal Ganglion Cell Health in Retinal Neurodegeneration

**DOI:** 10.4172/2155-9899.1000188

**Published:** 2014-02

**Authors:** Cathryn R Formichella, Simone K Abella, Stephanie M Sims, Heather M Cathcart, Rebecca M Sappington

**Affiliations:** 1 Department of Ophthalmology and Visual Sciences, Vanderbilt University School of Medicine, Nashville, Tennessee, USA; 2 Department of Pharmacology, Vanderbilt University School of Medicine, Nashville, Tennessee, USA

**Keywords:** Neuroinflammation, Glia, Aging, Astrocytes, Glaucoma, DBA/2, Axon transport

## Abstract

Retinal ganglion cell (RGC) loss in glaucoma is sectorial in nature and preceded by deficits in axonal transport. Neuroinflammation plays an important role in the pathophysiology of glaucoma in the retina, optic nerve and visual centers of the brain, where it similarly appears to be regulated spatially. In a murine model, we examined the spatial characteristics of astrocyte reactivity (migration/proliferation, hypertrophy and GFAP expression) in healthy retina, retina with two glaucoma-related risk factors (aging and genetic predisposition) and glaucomatous retina and established relationships between these reactivity indices and the spatial organization of astrocytes as well as RGC health. Astrocyte reactivity was quantified by morphological techniques and RGC health was determined by uptake and transport of the neural tracer cholera toxin beta subunit (CTB). We found that: (1) astrocyte reactivity occurs in microdomains throughout glaucomatous retina as well as retina with risk factors for glaucoma, (2) these astrocyte microdomains are primarily differentiated by the degree of retinal area covered by the astrocytes within them and (3) percent retinal area covered by astrocytes is highly predictive of RGC health. Our findings suggest that microdomains of astrocyte reactivity are biomarkers for functional decline of RGCs. Based on current and emerging imaging technologies, diagnostic assessment of astrocytes in the nerve fiber layer could succeed in translating axonal transport deficits to a feasible clinical application.

## Introduction

Glaucoma is characterized by progressive neurodegeneration of retinal ganglion cells (RGCs) and their axons, which form the optic nerve. As in many neurodegenerative disorders, glaucomatous loss of RGCs is preceded by deficits in axonal transport [1–8]. Axonal transport deficits and subsequent loss of RGCs and their axons occur in a sectorial fashion, where a glaucomatous retina will contain areas of healthy, dysfunctional and degenerating RGCs [2,3,9,10]. Glial reactivity and neuroinflammation also play a key role in glaucoma pathophysiology, which is evident in the retina, optic nerve and brain [7,11–24]. Recent evidence suggests that like RGC pathology, neuroinflammation is also spatially-regulated [17,19,24]. Here we assessed the spatial characteristics of astrocyte reactivity in a murine model of chronic glaucoma (DBA/2 mice), using morphological techniques, and correlated the spatial organization and reactivity of astrocytes with RGC uptake and transport of the axonal tracer, cholera toxin beta subunit (CTB). We examined a total of four experimental groups: healthy retina (young C57 mice), glaucomatous retina (aged DBA/2 mice) and retina with two different risk factors for glaucoma: normal aging (aged C57 mice) and a genetic predisposition to glaucoma (young DBA/2 mice). We found that: (1) astrocyte reactivity occurs in microdomains throughout glaucomatous retina as well as retina with risk factors for glaucoma, (2) these astrocyte microdomains are primarily differentiated by the degree of retinal area covered by the astrocytes within them and (3) percent retinal area covered by astrocytes is highly predictive of RGC health. Based on current and emerging imaging technologies, clinical assessment of astrocyte coverage is feasible. Therefore, astrocyte reactivity has the potential to be a biomarker for decreased RGC health that could broaden the therapeutic window for glaucoma patients by allowing practitioners to identify regions with faltering RGC health prior to overt nerve fiber layer or visual field defects.

## Materials and Methods

### Animals and tissue preparation

The experimental procedures described were approved by the Vanderbilt University Medical Center Institutional Animal Care and Use Committee. Retina from young (2–4 month, n=8) and aged (10–12 month, n=12) DBA/2 mice were compared to age-matched C57BL/6 mice (Jackson Laboratories, n=17). The average IOP for DBA/2 mice was 15 mmHg ± 1.4 mmHg and 22 mmHg ± 5.9 mmHg for young and aged mice, respectively. IOP was monitored monthly in awake, behaving DBA/2 mice using a tonometer (Tonolab) before mice were sacrificed [24].

### Neural tracing

Anterograde transport tracing of the retino-collicular projection was performed as previously described [3]. Briefly, 1 μl of 1% cholera toxin beta subunit conjugated to Alexa Fluor-488 or -594 (CTB; in sterile PBS solution; Invitrogen) was delivered bilaterally via a single, intravitreal injection, using a Hamilton syringe. Three days after CTB injection, mice were sacrificed by trans-cardial perfusion with 4% paraformaldehyde (PFA), eyes enucleated and brain removed for 24 hour post-fixation in 4% PFA.

### Whole mount retina immunohistochemistry

Immunohistochemistry in whole mount retina was performed as previously described [17,24,25]. Briefly, retina were cryoprotected in a graded sucrose series (10–30%) and then freeze-thawed on dry ice. Autofluorescence was quenched with 0.1% sodium borohydride (Fisher) solution for 30 minutes at room temperature. Subsequently, retinas were rinsed with PBS and incubated in a blocking solution of 5% normal horse serum (NHS; Sigma-Aldrich; St. Louis, MO) and 0.1% Triton X-100 (Fisher) in PBS overnight at 4°C. Following the blocking step, retinas were transferred into a primary antibody solution containing: mouse anti-GFAP (1:500; catalog number MAB360, EMD Millipore Corporation; Billerica, MA), 3% NHS and 0.1% Triton X-100 in PBS for 4 days at 4°C. After rinsing in PBS, the retinas were incubated overnight at room temperature with a secondary antibody solution, containing donkey anti-mouse Alexa-Fluor 647 (7.5 μg/ml; Jackson Immuno Research; West grove, PA), 1% NHS and 0.1% Triton X-100. Retinas were rinsed, mounted on glass slides with Fluoromount-G (Fisher) and cover-slipped.

### Quantitative fluorescence microscopy (Retina)

Whole mount retina imaging was performed through the Vanderbilt University Medical Center Cell Imaging Shared Resource Core. Staining was imaged on an upright confocal microscope (LSM510 META; Carl Zeiss Inc., Thornwood, NY) or an inverted confocal microscope (Olympus FV-1000; Center Valley, PA) equipped with laser scanning fluorescence (blue/green, green/red, red/far-red). Three dimensional z-series images of the retina were acquired using a digital camera and image analysis software (LSM5; Zeiss or FV-10 ASW; Olympus). For each retina, 5–10 pseudorandom images through the ganglion cell and nerve fiber layers were obtained at 63× magnification.

### Quantification of digital microscopy

For each of the 5–10 pseudorandom retinal images the ganglion cell- and nerve fiber-layers were collapsed into two dimensional images. We used these images to examine astrocyte reactivity and CTB labeling intensities. Images were collected between the mid-peripheral to mid-central regions of the retina; thereby avoiding the optic disc and the area immediately surrounding the optic disc, which contains large bundles of RGC axons and higher density of astrocytes that would confound our studies of CTB uptake in RGC soma as well as accuracy of astrocyte measurements. All measurements were performed using imaging analysis software (Image Pro Plus 5.1; Media Cybernetics, Inc; Bethesda, MD or ImageJ, NIH). Measurements were made by two different examiners that were blind to experimental parameters.

#### Astrocyte and RGC density

To evaluate changes in the density of microglia, we hand-counted the number of GFAP+ astrocyte cell somas and CTB+ RGC cell somas in each micrograph from DBA/2 and C57 retina. We divided soma counts by the area of the micrograph to calculate the number of GFAP+ astrocytes and CTB+ RGCs per mm^2^ of retina. Hand-counts were performed by an experimenter blind to the experimental groups and the data were analyzed by a second experimenter. Density data are presented as astrocytes/mm^2^ and CTB+ RGCs/mm^2^.

#### Astrocyte hypertrophy

To evaluate hypertrophy, we measured the soma size of astrocytes by hand-tracing of each GFAP+ cell soma in each micrograph. Soma area was determined as the area contained within the soma trace. Hand-tracing was performed by an experimenter blind to the experimental groups and the data were analyzed by a second experimenter. Hypertrophy data are presented as the soma area in mm^2^.

#### GFAP and CTB intensity

GFAP and CTB intensity was calculated as the mean pixel intensity across each image. This value represents the total intensity values used for astrocyte and RGC health studies. For astrocyte reactivity studies, this value was divided by the number astrocytes in the image to yield mean GFAP intensity/cell.

#### % GFAP coverage

To create one index that represented changes in both astrocyte density and hypertrophy, we utilized image analysis software (ImageJ) to count the number of pixels with GFAP label above background for each micrograph. The number of GFAP+ pixels was divided by the total number of pixels in the image and expressed as a percentage (% GFAP coverage).

### Quantification of axonal transport (Superior Colliculus)

Brains were incubated in a sucrose series (10%–20%–30%), cortex removed and 50 μm-thick serial, coronal sections through the superior colliculus were obtained on a sliding microtome. Sections were mounted serially on slides CTB signal was photographed digitally on an inverted fluorescent microscope (Eclipse Ti; Nikon Instruments; Melville, NY). Using layer IV as the ventral border, we outlined the boundaries of the superficial superior colliculus, which was partitioned in to 6 μm bins from medial to lateral. CTB density was calculated as the area of pixels with CTB labeling above background (pixel strength of the non-retinorecipient layers of the superior colliculus) divided by the total pixel area for sections for each 6 μm bin. Using a colorimetric scale to illustrate % CTB density in each section, we constructed a colorimetric representation of CTB density across the retinotopic colliculus map, which was based on section thickness and intersection distance. For each SC, we determined the fraction of intact retinotopic map, defined as the percent area with CTB signal ≥ 70% density. The mean% transport was used for statistical analysis.

### Statistical analysis

All statistical tests were performed with SigmaPlot (Systat Software Inc., San Jose, CA). For comparison of means between conditions, we used Kruskal-Wallace one-way analysis of variance followed by post hoc pair wise comparisons between groups using Dunn’s Method. Correlations were assessed by both Pearson Product Moment Correlation and Spearman’s Rank Order Correlation. Statistically significant correlations were verified by polynomial regression analysis. Error bars represent ± SD of the mean. For all analyses, *P* ≤ 0.05 was considered statistically significant.

## Results

### Astrocyte morphology is spatially variable within individual retina, particularly those challenged by glaucoma-related stressors

RGC neurodegeneration, microglia reactivity and IL-6 signaling in glaucoma occur in a spatially-dependent manner [2,3,15,17,19,24]. Given that astrocytes are well-established as additional mediators of neuroinflammation and RGC health in glaucoma, we examined whether astrocyte reactivity is also spatially regulated. For these studies, we utilized the DBA/2 mouse model of inherited glaucoma, where elevations in IOP induce neurodegeneration of RGCs and their axons [2,9,10,26,27]. Elevated IOP in these mice results from mutations in two genes (gpnmb and tyrp1) and develops gradually with age, much like the human form of the disease [28–32]. Combined with age-matched controls, the age-dependent nature of RGC degeneration in this model provides a unique opportunity to compare two significant risk factors for glaucoma, aging (aged C57) and genetic susceptibility (young DBA/2), to healthy (young C57) and disease (aged DBA/2) states.

**Figure 1:**
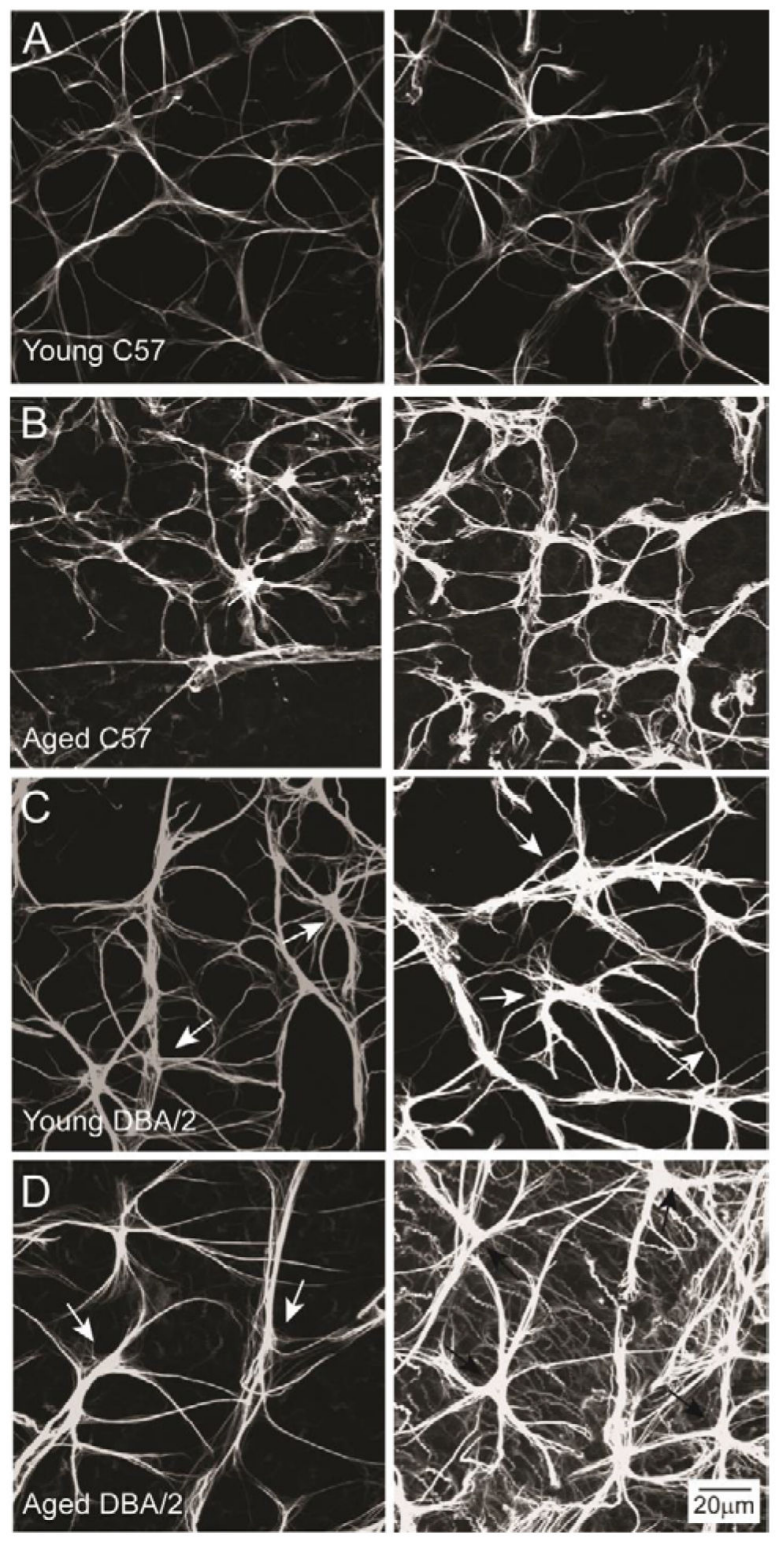
Astrocyte morphology is variable across individual retina. Representative confocal micrographs of whole-mount retina from young C57 (A), aged C57 (B), young DBA/2 (C) and aged DBA/2 (D) mice immunolabeled with the astrocyte marker GFAP. Left and right columns illustrate variations in astrocyte morphology from comparable retinal areas within an individual retina. Glaucomatous retina (aged DBA/2) exhibits a broader range of astrocyte morphologies than young C57, aged C57 and young DBA/2 retina. Scale is the same for (A–D).

We first performed a qualitative analysis of astrocyte reactivity in wholemounted retina immunolabeled with the astrocyte marker glial fibrillary acidic protein (GFAP; Figure 1). GFAP is an intermediate neurofilament that is expressed by all astrocytes and is upregulated in disease states that involve hypertrophy of astrocytes. In retina, astrocytes are confined to the nerve fiber layer, which is the inner most layer of the retina that houses RGC axons and large caliber blood vessels that supply the inner retina. Astrocytes are oriented in a monolayer that surrounds RGC axons and blood vessels and is perpendicular to the rest of the neural retina.

Qualitative assessment of astrocyte morphology across 200 μm×200 μm fields of retina from young C57, aged C57, young DBA/2 and aged DBA/2 retina revealed that, in similar areas of retinal eccentricity, there is rather large variability in the morphology of astrocytes within individual retina, especially those with glaucoma or glaucoma-related stressors (Figures 1A–1D). Not surprisingly, GFAP expression appeared stronger on a per cell basis in aging C57, young DBA/2 and aged DBA/2 retina than in young, healthy C57 retina (Figures 1B–1D verses 1A). In glaucomatous retina (aged DBA/2), GFAP expression was especially variable with some areas exhibiting moderate GFAP expression and other areas of the same retina exhibiting very strong GFAP expression (Figure 1D). The same was true for density, where aged DBA/2 retina also exhibited the greatest intra-retinal variability (Figure 1D). Although less obvious than astrocyte density and GFAP expression, the size of individual astrocytes also varied dramatically, particularly in young and aged DBA/2 retina (arrows; Figures 1C and 1D). Given that upregulation of GFAP, hypertrophy and increased density are all hallmarks of astrocyte reactivity, these data suggest that astrocyte reactivity may occur in a spatially-dependent manner in retina challenged by glaucoma-related stressors.

### Neurodegenerative stressors do not alter the mean density of astrocytes in retina, but instead alter how astrocytes are spatially distributed

To quantify the apparent variability in astrocyte morphology related to reactivity, we quantitatively measured three indices of reactivity, including astrocyte proliferation/migration (density), hypertrophy and GFAP expression. Measurements were obtained from a minimum of ten fields per retina and the mean across images was used to determine overall changes in reactivity indices, while histogram functions were used to assess the distribution (spatial variability) of reactivity measures for each experimental group.

**Figure 2:**
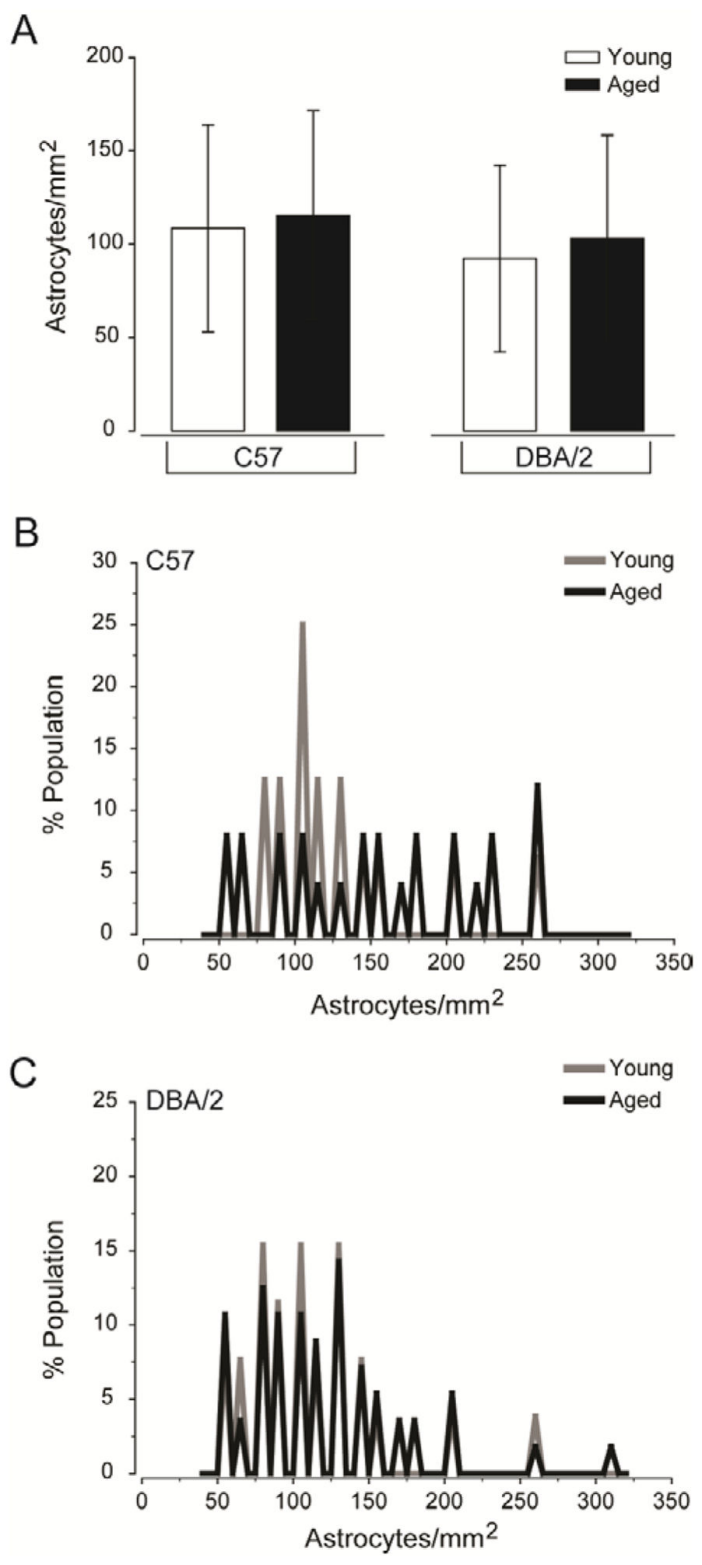
Glaucoma-related stressors alter astrocyte distribution rather than total density. (A) Quantification of the mean density of astrocytes (astrocytes/mm^2^; y-axis) across all images and samples reveals no difference in the overall density of astrocytes in retina challenged by glaucoma-related stressors, as compared to healthy retina. Error bars indicate standard deviation and asterisks indicate p<0.05. (B) Histogram function of astrocyte density for young C57 (gray) and aged C57 (black) retina plotted as percent population (y-axis) versus astrocytes/mm^2^ (x-axis). The histogram functions reveal a larger spread in the distribution of astrocyte densities in aged C57 retina, as compared to healthy retina. (C) Histogram function of astrocyte density for young DBA/2 (gray) and aged DBA/2 (black) retina plotted as percent population (y-axis) versus astrocytes/mm^2^ (x-axis). The histogram functions reveal a larger spread in both distributions, as compared to young C57 retina (B) However, aged DBA/2 retina exhibits a larger spread in the distribution than young DBA/2 retina.

For analysis of astrocyte proliferation/migration, we hand-counted GFAP+ astrocytes and calculated the density for young and aged C57 and DBA/2 retina. The mean astrocyte density in retina was similar for all groups (p>0.05; Figure 2A). Although the mean density of astrocytes did not differ, histogram functions for astrocyte density revealed that the distribution of densities that contributed to the means differed dramatically (Figures 2B and 2C). In general, retina exposed to glaucoma-related stressors, including normal aging, exhibited a wider distribution of astrocyte densities than healthy, young C57 retina (Figures 2B and 2C). In young C57 retina, astrocytes densities were clustered between 75 and 120 astrocytes/mm^2^ (Figure 2B). In contrast, astrocyte densities ranged from 50 to 220 astrocytes/mm^2^ in aged C57 retina (Figure 2B). In retina with glaucoma-specific stressors (DBA/2), the range of astrocyte densities represented increased with severity of the stressor. In young DBA/2 retina, astrocyte densities ranged from 50 to 260 astrocytes/mm^2^ (Figure 2C). In glaucomatous, aged DBA/2 retina, astrocyte densities ranged from 50 to 320 astrocytes/mm^2^ (Figure 2C). These data indicate that while the overall density of astrocytes in retina remains unchanged, neurodegenerative stressors alter the spatial distribution of astrocytes to produce areas with lower astrocyte density and areas with higher astrocyte density. Of particular note, increased severity of the stressor tended to increase the maximum density of astrocytes contained within microdomains of retina.

### Astrocyte hypotrophy is a common characteristic of retina exposed to risk factors for glaucoma

**Figure 3:**
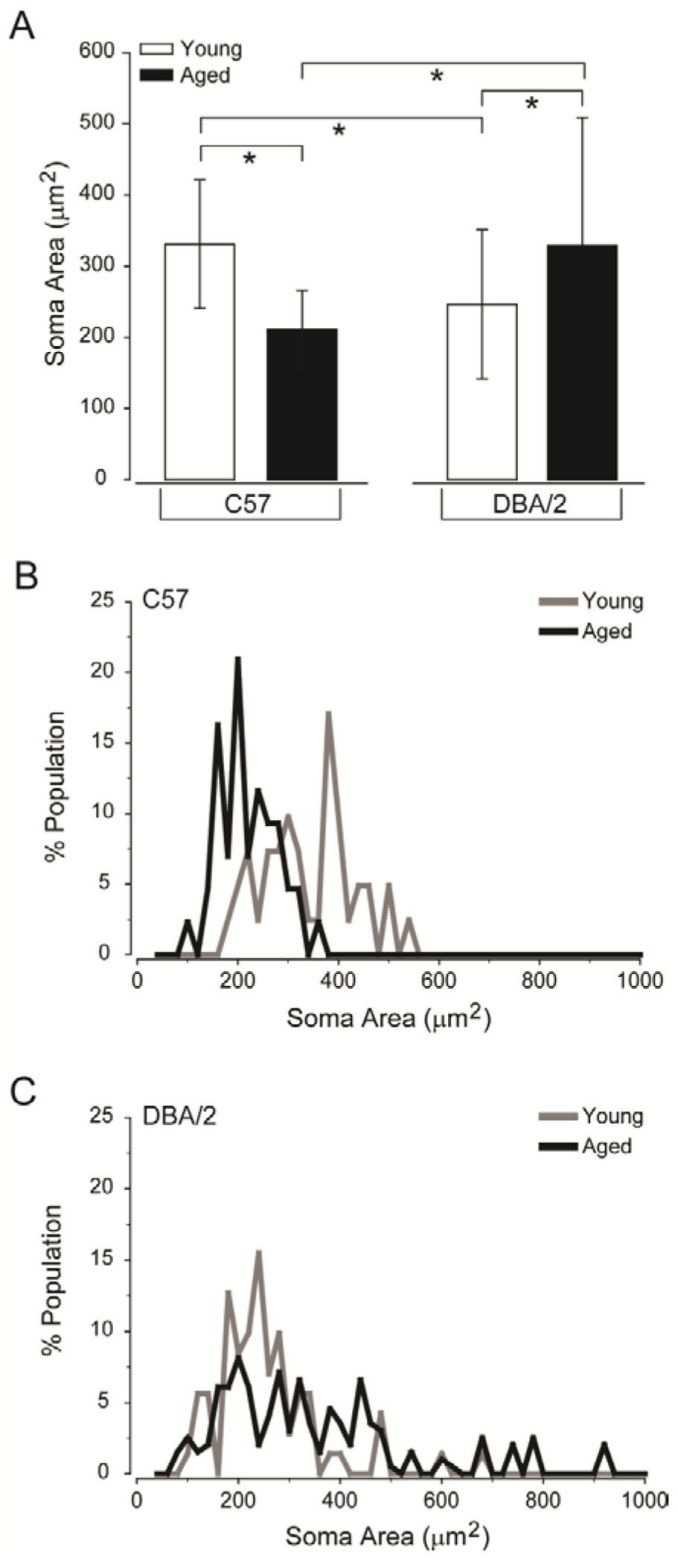
Glaucoma risk factors primarily induce astrocyte hypotrophy, while glaucoma induces greater variability in astrocyte size. (A) Quantification of the mean soma area of astrocytes (μm^2^; y-axis) across all images and samples reveals that the soma of astrocytes in aged C57 and young DBA/2 retina are smaller than that of young C57 and aged DBA/2 retina. Error bars indicate standard deviation and asterisks indicate p<0.05. (B) Histogram function of soma area for young C57 (gray) and aged C57 (black) retina plotted as percent population (y-axis) versus soma area (μm^2^; x-axis). The histogram functions reveal a reduction in the distribution spread and left shift towards smaller soma areas in aged C57, as compared to young C57 retina. (C) Histogram function of soma area for young DBA/2 (gray) and aged DBA/2 (black) retina plotted as percent population (y-axis) versus soma area (μm^2^; x-axis). The histogram functions for young and aged DBA/2 retina reveal an increase in the distribution spread, as compared to young C57 (B) retina. Aged DBA/2 retina exhibits the largest spread that lacks a primary peak, while young DBA/2 retina exhibits primary peaks that are shifted towards smaller soma areas.

To measure hypertrophy of astrocytes, we hand-traced GFAP+ cell somas and calculated the cytosolic area contained within the traced perimeter. Quantification revealed that the mean soma area of astrocytes was similar in young C57 and aged DBA/2 retina (p>0.05) as well as in aged C57 and young DBA/2 retina (p>0.05; Figure 3A). However, aged C57 and young DBA/2 retina contained astrocytes with soma areas 25–35% smaller than young C57 and aged DBA/2 retina (p<0.05 for all; Figure 3A). In addition to population-wide shifts in astrocyte soma area, histogram analysis revealed marked differences in the distribution of soma areas, even among groups with similar means of soma area (Figures 3B and 3C). Aged C57 retina exhibited a left shift in soma area as well as a tighter overall distribution, as compared to young C57 retina (Figure 3B). This suggests an overall trend towards astrocyte hypotrophy that encompasses the entire astrocyte population. In contrast, DBA/2 retina, regardless of age, exhibited spreading of soma area distribution, as compared to both young and aged C57 retina (Figures 3B and 3C). Like aged C57 retina, young DBA/2 retina exhibited a left shift in soma area distribution for a majority of astrocytes, indicating a tendency for astrocyte hypotrophy (Figure 3C compare to 3B). However, unlike aged C57 retina, young DBA/2 retina also exhibited spreading of the soma area distribution to the right with maximum soma area around 600 μm (Figure 3C). This indicates that although the overall trend is towards astrocyte hypotrophy in young DBA/2 retina, there is a small percentage of astrocytes that undergo hypertrophy. Aged DBA/2 retina exhibited the greatest spread in the distribution of astrocyte soma area, which ranged from 100 μm to over 900 μm (Figure 3C). The distinct absence of a primary peak for the distribution curve indicates significant variability in soma area across the astrocyte population (Figure 3C). These data suggest that glaucomatous neurodegeneration induces marked variation in astrocyte hypertrophy, while high risk conditions for glaucoma, like normal aging and genetic predisposition, induce a striking tendency for astrocyte hypotrophy.

### GFAP per cell is generally decreased in retina challenged by neurodegenerative stressors

**Figure 4:**
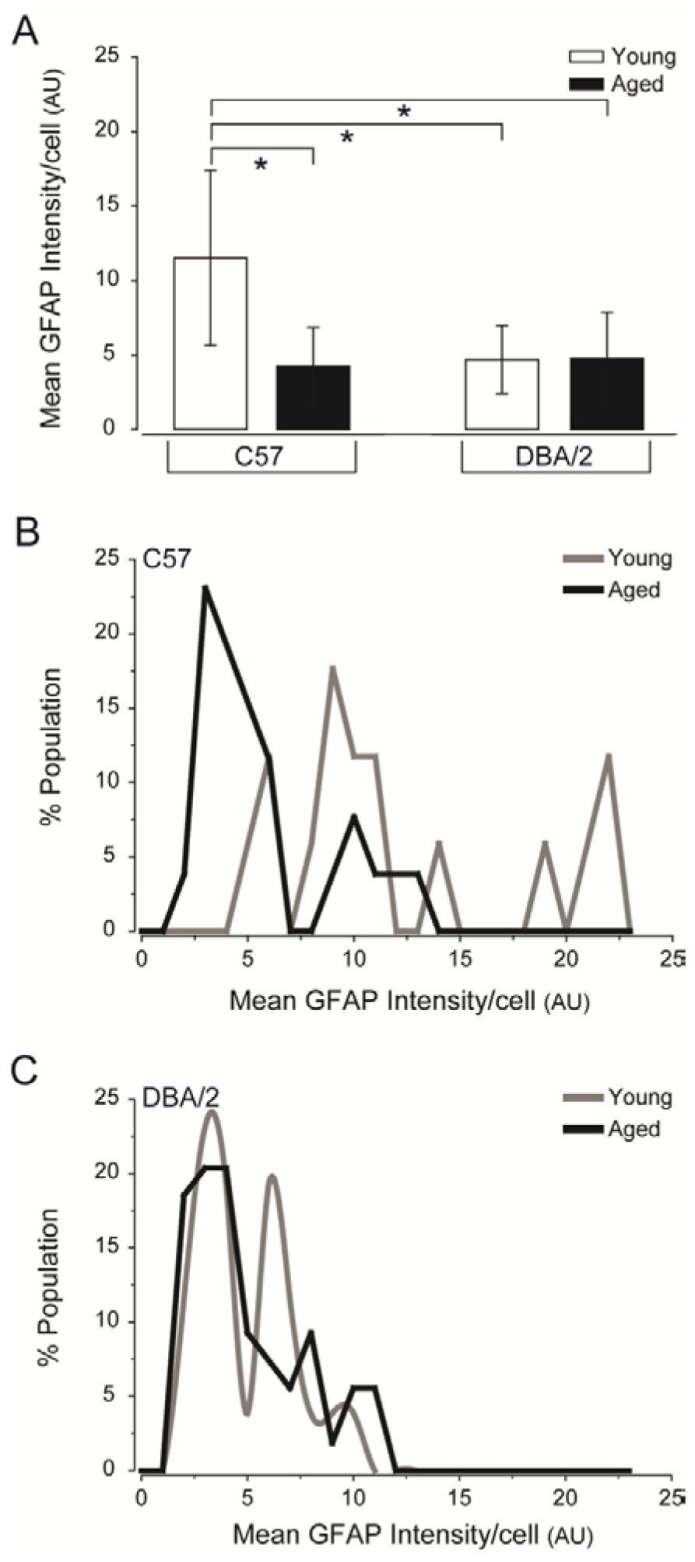
Decreased GFAP expression/cell is a common characteristic of retina challenged by glaucoma-related stressors. (A) Quantification of the mean GFAP intensity/cell (arbitrary units; y-axis) across all images and samples reveals that mean GFAP intensity/cell is higher in young C57 retina than in young DBA/2, aged C57 and aged DBA/2 retina. Error bars represent ± standard deviation and asterisks indicate p<0.05. (B) Histogram function of GFAP intensity/cell for young C57 (gray) and aged C57 (black) retina plotted as percent population (y-axis) versus GFAP intensity/cell (arbitrary units, x-axis). The histogram function for aged C57 retina reveals a shift towards lower GFAP intensity and a smaller range of distribution, as compared to young C57 retina. (C) Histogram function of GFAP intensity/cell for young DBA/2 (gray) and aged DBA/2 (black) retina plotted as percent population (y-axis) versus GFAP intensity/cell (arbitrary units; x-axis). The histogram function for young DBA/2 and aged DBA/2 retina demonstrate a similar range of distributions that is shifted towards lower GFAP expression. However, young DBA/2 retina exhibits the presence of two distinct peaks of GFAP expression that account for almost 50% of GFAP expression in the population, while aged DBA/2 retina exhibits a single peak accounting for only 20% of astrocyte population.

Finally, we quantified GFAP expression by measuring the total intensity of GFAP immunofluorescence in each confocal micrograph. To account for astrocyte density, we divided the total intensity of GFAP immunofluorescence by the number of astrocytes in each micrograph. This yielded the average GFAP expression per cell, which was then used for all subsequent analyses. We found that GFAP expression by astrocytes was approximately 60% higher in young C57 retina than in aged C57, young DBA/2 and aged DBA/2 retina (p<0.05 for all; Figure 4A). There was no significant difference in GFAP intensity/cell between aged C57, young DBA/2 and aged DBA/2 retina (p>0.05 for all; Figure 4A). Histogram analysis revealed that the distribution of GFAP expression per cell was quite similar in retina challenged by neurodegenerative stressors, which all exhibited a marked left shift towards lower intensity levels, as compared to that of young C57 retina (Figures 4B and 4C). The distribution of GFAP expression by astrocytes was most similar between aged C57 and aged DBA/2 retina, suggesting a potential age-related etiology for decreased GFAP expression (Figures 4B and 4C). Unlike aged C57 and aged DBA/2 retina, young DBA/2 retina exhibited two primary peaks of GFAP expression, indicating a distinct division of the astrocyte population with regard to level of GFAP expression (Figure 4C). These data suggest that an overall reduction in GFAP expression per cell is a common characteristic of astrocytes in retina challenged by neurodegenerative stressors and that age may be a significant factor in determining level of expression.

### Reactivity indices identify the presence of distinct astrocyte microdomains that increase in number and diversity in retina challenged by neurodegenerative stressors

**Figure 5:**
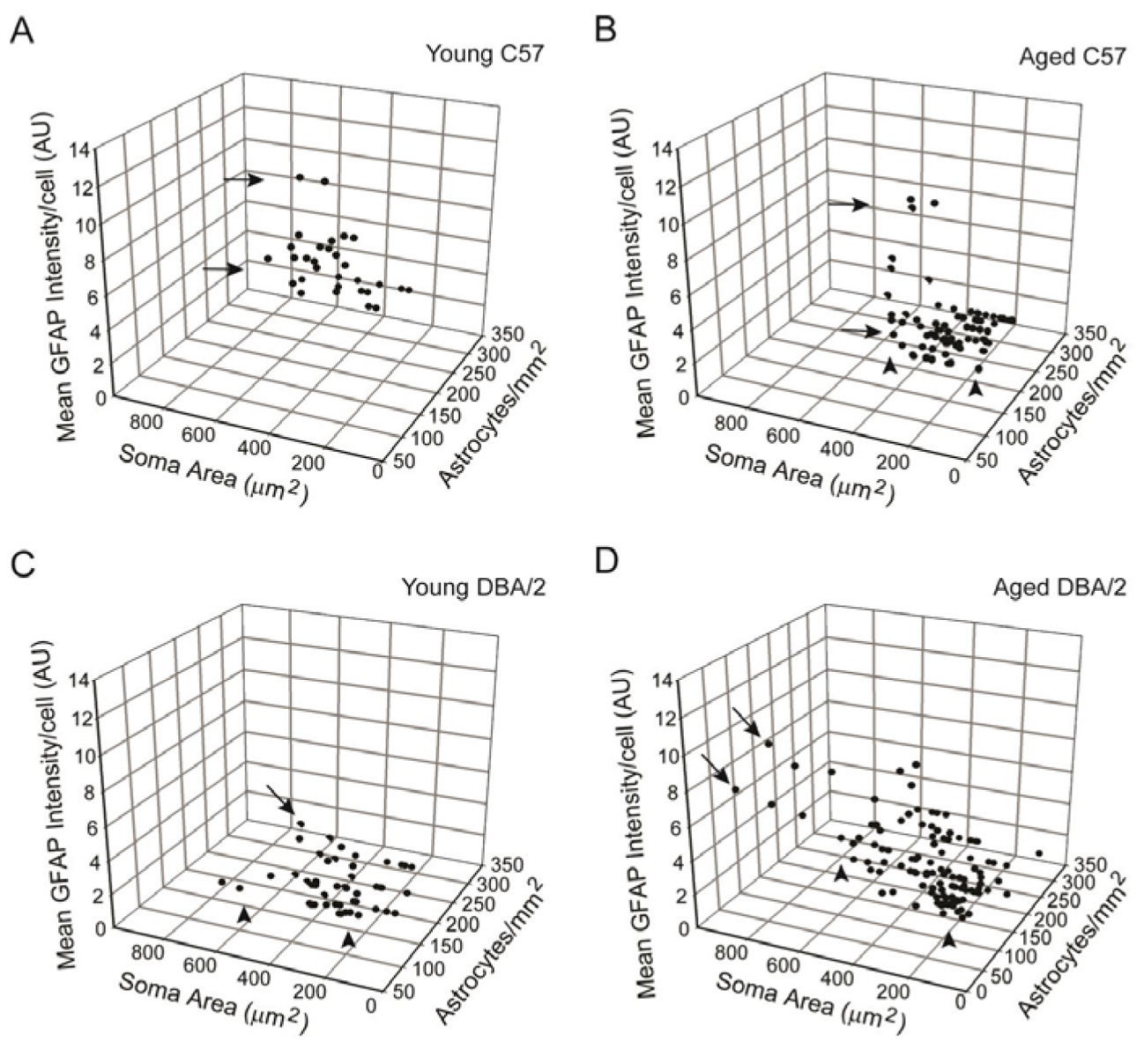
Spatially-coincident astrocytes share morphological characteristics that suggest the presence of distinct microdomains of astrocyte reactivity. Three-dimensional plots of spatially-coincident reactivity indices in young C57 (A), aged C57 (B), young DBA/2 (C) and aged DBA/2 (D) retina, where each data point represents measurements obtained from a single micrograph. For all graphs, reactivity indices are represented as: astrocytes/mm^2^ (y-axis), soma area (x-axis) and mean GFAP intensity/cell number (z-axis). (A) In young C57 retina, astrocytes exhibit similar morphological attributes across the population, with only some variability noted in GFAP intensity/cell (arrows). (B) In aged C57 retina, morphological attributes of spatially-coincident astrocytes become more variable, with distinct microdomains emerging with respect to density, GFAP intensity/cell (arrows) and soma area (arrowheads). (C) Young DBA/2 retina exhibits variability in morphological attributes in spatially-coincident astrocytes that is greater than that observed in young C57 retina, but less than that of aged C57 retina. Distinct microdomains appear with respect to density, GFAP intensity/cell (arrows) and soma area (arrowheads). (D) Aged DBA/2 retina exhibits the largest number and diversity of microdomains with significant variability in density, GFAP intensity/cell (arrows) and soma area (arrowheads).

To determine whether variations in reactivity indices for astrocytes are spatially-related, we measured and graphed all reactivity indices across ten confocal micrographs from each retina analyzed (Figure 5). For these graphs, each data point represents the measurements obtained from one confocal micrograph. The resulting 3-dimensional plots revealed substantial differences in the relationships between reactivity indices and identified spatially discreet populations of astrocytes that differ in reactivity state. In healthy retina from young C57 mice, spatially discreet populations of astrocytes exhibit similar reactivity states, as evidenced by the relatively tight clustering of the data points (Figure 5A). Most of the spread in the data points was relegated to the y-axis, suggesting that variation in the astrocyte population of healthy retina is most attributable to soma area (Figure 5A). In contrast, aged C57 retina exhibited marked spreading of the data points along the y-axis and z-axis (Figure 5B). Clustering of data points indicate possible relationships between soma area (y-axis) and density (x-axis), where astrocytes with larger soma appear to reside together in lower density than those with smaller soma (arrowheads; Figure 5B). Similarly, these larger astrocytes also appear to have a higher level of GFAP expression (y-axis; arrows; Figure 5B). Similar to aged C57 retina, young DBA/2 retina exhibited spreading of the data points with a tendency for astrocytes with larger soma appear to reside together in lower density than those with smaller soma (arrowheads; Figure 5C). Although to a lesser degree than in aged C57 retina, astrocytes in young DBA/2 retina with larger soma also appeared to have a higher level of GFAP expression (y-axis; arrows; Figure 5C). Finally, aged DBA/2 retina exhibited the greatest degree of spreading and therefore, variability in the morphological characteristics of astrocytes (Figure 5D). Again, astrocyte soma area and density appeared to be interrelated in aged DBA/2 retina (Figure 5D). However, in aged DBA/2 retina, astrocytes with larger soma appeared to reside together at higher densities rather than lower densities, as observed in aged C57 and young DBA/2 retina (arrowheads; Figure 5D compare to 5B and 5C). Aged DBA/2 retina also exhibited the trend for astrocytes with larger soma to have higher GFAP expression (Figure 5D). Together, these data suggest that astrocyte reactivity likely occurs in microdomains, which have distinct reactivity profiles that increase in both number and diversity with severity of the stressor.

### Astrocyte microdomains can be reliably predicted in retina

**Figure 6:**
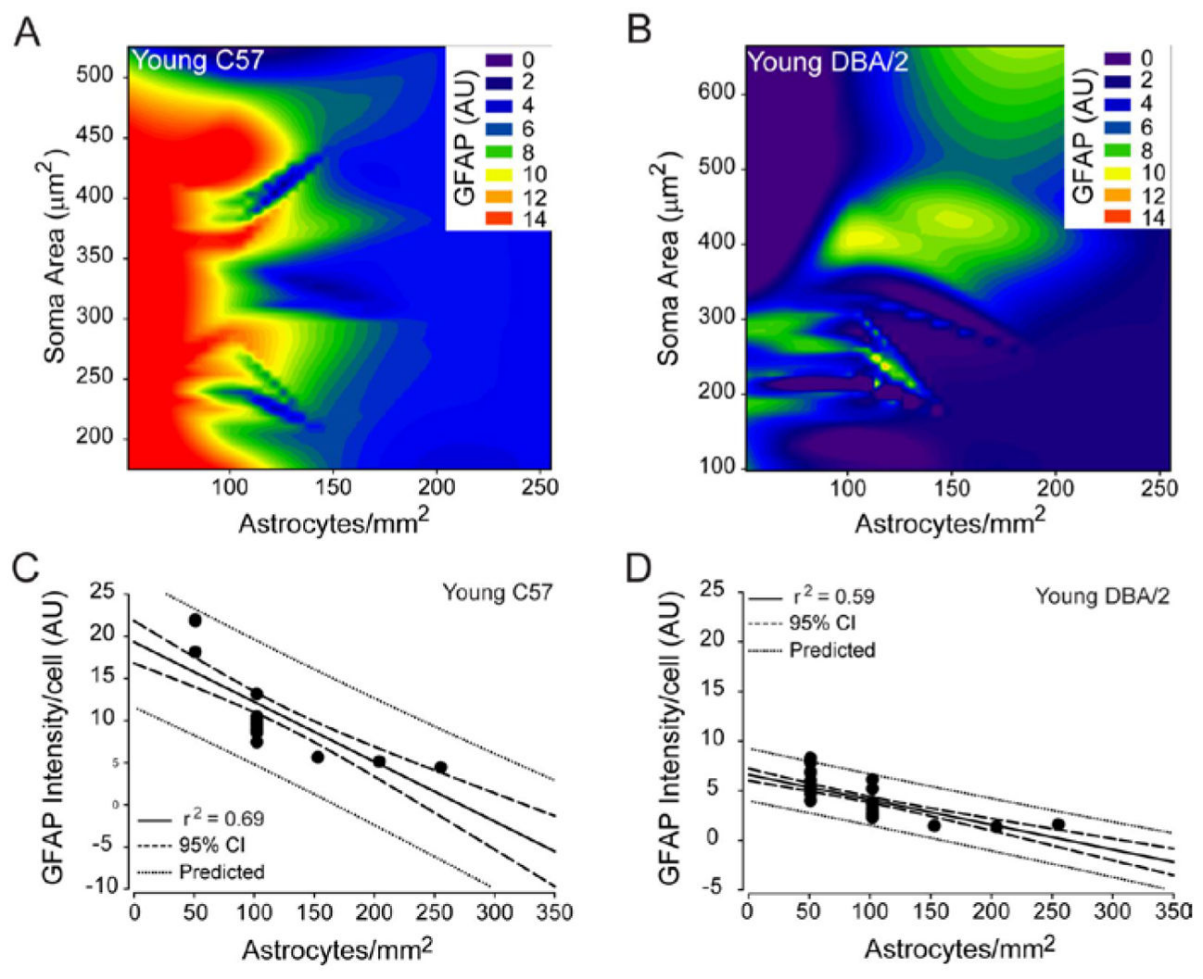
Astrocyte density reliably predicts GFAP expression by spatially-coincident astrocytes in young C57 and young DBA/2 retina. (A and B) Contour plot depicting the relationships between mean GFAP intensity/cell (color bar; arbitrary units), astrocyte density (x-axis; astrocytes/mm^2^) and soma area (y-axis; μm^2^) in young C57 (A) and young DBA/2 (B) retina. (C) Regression graph of the negative correlation between astrocyte density and mean GFAP expression/cell in young C57 retina plotted as astrocytes/mm^2^ (x-axis) versus arbitrary units of intensity (y-axis). Solid lines indicate regression line based on r^2^ value. Dashed lines indicate the 95% confidence interval. Dotted lines indicate predicted values based on r^2^ and 95% confidence. (D) Regression graph of the negative correlation between astrocyte density and mean GFAP expression/cell in young DBA/2 retina plotted as astrocytes/mm^2^ (x-axis) versus arbitrary units of intensity (y-axis). Solid lines indicate regression line based on r^2^ value. Dashed lines indicate the 95% confidence interval. Dotted lines indicate predicted values based on r^2^ and 95% confidence.

Our morphological analyses suggest that spatially-coincident astrocytes share similar morphological features that could identify the presence of distinct astrocyte microdomains. To determine whether astrocyte microdomains truly exist in retina or whether they are an epiphenomenon of random variability in reactivity states, we examined the predictability of reactivity indices in spatially-coincident astrocytes, using correlation and polynomial regression analyses. Astrocyte density was negatively correlated with GFAP intensity/cell in young C57 (coeff=−0.793, p<0.01; Figure 6A) and young DBA/2 retina (coeff=−0.755, p<0.01; Figure 6B) and decreased Iba-1 intensity/cell (coeff=−0.80, p<0.01). This relationship was highly predictable for both young C57 (r^2^=0.63, p<0.01) and young DBA/2 retina (r^2^=0.59, p<0.01), where astrocyte density could accurately predict GFAP expression in 63% and 59% of microdomains, respectively (Figures 6C and 6D). There was no significant correlation between soma area and either GFAP expression or astrocyte density in young C57 and young DBA/2 retina (p>0.05).

**Figure 7:**
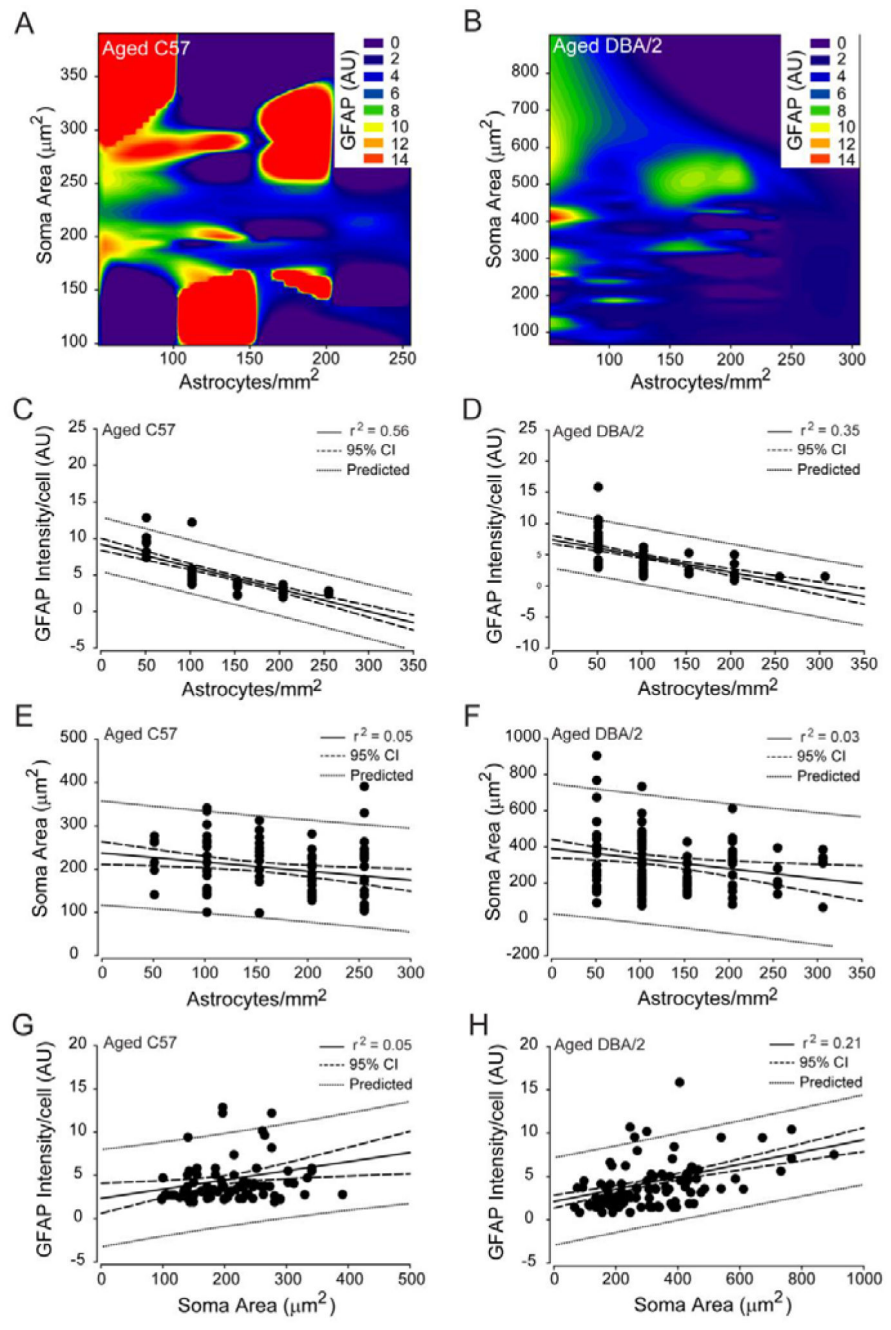
Density, hypertrophy and GFAP expression for spatially-coincident astrocytes are reliably predictable in aged C57 and aged DBA/2 retina. (A and B) Contour plot depicting the relationships between mean GFAP intensity/cell (color bar; arbitrary units), astrocyte density (x-axis; astrocytes/mm^2^) and soma area (y-axis; μm^2^) in aged C57 (A) and aged DBA/2 (B) retina. (C) Regression graph of the negative correlation between astrocyte density and mean GFAP expression/cell in aged C57 retina plotted as astrocytes/mm^2^ (x-axis) versus arbitrary units of intensity (y-axis). (D) Regression graph of the negative correlation between astrocyte density and mean GFAP expression/cell in aged DBA/2 retina plotted as astrocytes/mm^2^ (x-axis) versus arbitrary units of intensity (y-axis). (E) Regression graph of the negative correlation between astrocyte density and soma area in aged C57 retina plotted as astrocytes/mm^2^ (x-axis) versus soma area (μm^2^; y-axis). (F) Regression graph of the negative correlation between astrocyte density and soma area in aged DBA/2 retina plotted as astrocytes/mm^2^ (x-axis) versus soma area (μm^2^; y-axis). (G) Regression graph of the positive correlation between soma area and mean GFAP intensity/cell in aged C57 retina plotted as soma area (μm^2^; sxaxis) versus arbitrary units of intensity (y-axis). (H) Regression graph of the positive correlation between soma area and mean GFAP intensity/cell in aged DBA/2 retina plotted as soma area (μm^2^; sxaxis) versus arbitrary units of intensity (y-axis). For all graphs, solid lines indicate regression line based on r^2^ value. Dashed lines indicate the 95% confidence interval. Dotted lines indicate predicted values based on r^2^ and 95% confidence.

In contrast to young C57 and young DBA/2 retina, astrocyte microdomains in aged C57 and aged DBA/2 retina could be predicted by relationships between all three reactivity indices. Like young C57 and young DBA/2 retina, astrocyte density was negatively correlated with GFAP intensity/cell in both aged C57 (coeff=−0.713, p<0.01; Figure 7A) and aged DBA/2 retina (coeff=−0.591, p<0.01; Figure 7B). This relationship had the highest predictive capacity across the reactivity indices, where astrocyte density could accurately predict GFAP expression in 56% and 35% of spatially-coincident astrocytes in aged C57 (r^2^=0.56, p<0.01) and aged DBA/2 retina (r^2^=0.35, p<0.01), respectively (Figures 7C and 7D). Of note, the predictive capacity of the negative relationship between astrocyte density and GFAP expression was greatly reduced in glaucomatous retina, as compared to the other conditions (Figure 7D). Astrocyte density also negatively correlated with soma area in aged C57 (coeff=−0.234, p=0.01) and aged DBA/2 retina (coeff=−0.195, p<0.01), respectively (Figures 7A and 7B). This tendency for microdomains with lower astrocyte density to contain more hypertrophied astrocytes was far less predictable than their tendency to have higher GFAP expression, as indicated by a predictive capacity of only 5% in aged C57 retina (r^2^=0.05, p=0.01; Figure 7E) and 3% in aged DBA/2 retina (r^2^=0.03, p=0.01; Figure 7F). In addition to astrocyte density, soma area of astrocytes was positively correlated with GFAP intensity/cell in both aged C57 (coeff=0.280, p<0.01) and aged DBA/2 retina (coeff=0.46, p<0.01; Figures 7A and 7B). This tendency for GFAP expression to be higher in hypertrophied astrocytes was much greater in aged DBA/2 retina than in aged C57 retina, where soma area could predict GFAP expression in 21% of DBA/2 astrocyte microdomains (r^2^=0.21, p<0.01; Figure 7H) versus only 5% in C57 microdomains (r^2^=0.05, p=0.01; Figure 7G). Together, these data indicate that the tendency for spatially-coincident astrocytes to share similar morphological characteristics results from reliable relationships between reactivity indices that are stressor-dependent and define distinct astrocyte microdomains.

### Variations in astrocyte morphology coincide with variations in RGC axonal transport

Astrocytes are known to influence RGC health in glaucoma through increased reactivity that leads to production of both neuroprotective and neurodestructive factors, including brain-derived neurotrophic factor, tumor necrosis factor alpha and nitric oxide [11]. Our data suggests that like other neuroinflammatory processes, astrocyte reactivity is spatially discreet and variable, yet predictable, across glaucomatous retina as well as retina with risk factors for glaucoma. Given that RGC degeneration in glaucoma occurs in a sectorial fashion, we examined whether astrocyte microdomains could align with spatial variations in RGC health. Using the well-documented neural tracer, cholera toxin beta subunit (CTB), we examined RGC health by quantifying axonal transport of CTB to the superior colliculus, the primary target of RGCs in the rodent visual system, as well as uptake of CTB in the retina. Deficits in axonal transport occur prior to structural loss of RGCs in glaucoma and exhibit the same sectorial pattern of pathology noted with axon, RGC soma and vision loss in glaucoma [3,10].

For these studies, CTB was delivered via intravitreal injection to the retina, where it is actively taken up by RGCs and actively transported (anterograde) to the superior colliculus. For axon transport studies, we sectioned the superior colliculus and measured CTB intensity in the retinorecipient layers of the superficial superior colliculus. CTB density was calculated as the area of pixels with CTB labeling divided by the total pixel area for sections at 6 μm intervals from medial to lateral borders of the superior colliculus. Using a colorimetric scale to illustrate % CTB density (0–100%) in each section, we constructed a colorimetric representation of CTB density across the retinotopic colliculus map, which was based on section thickness and intersection distance. CTB transport was analyzed as % transport. For CTB uptake studies, we examined both the total intensity of CTB and density of CTB+ RGCs micrographs from the mid-central to mid-peripheral region of retina from the same animals used in the superior colliculus studies.

**Figure 8:**
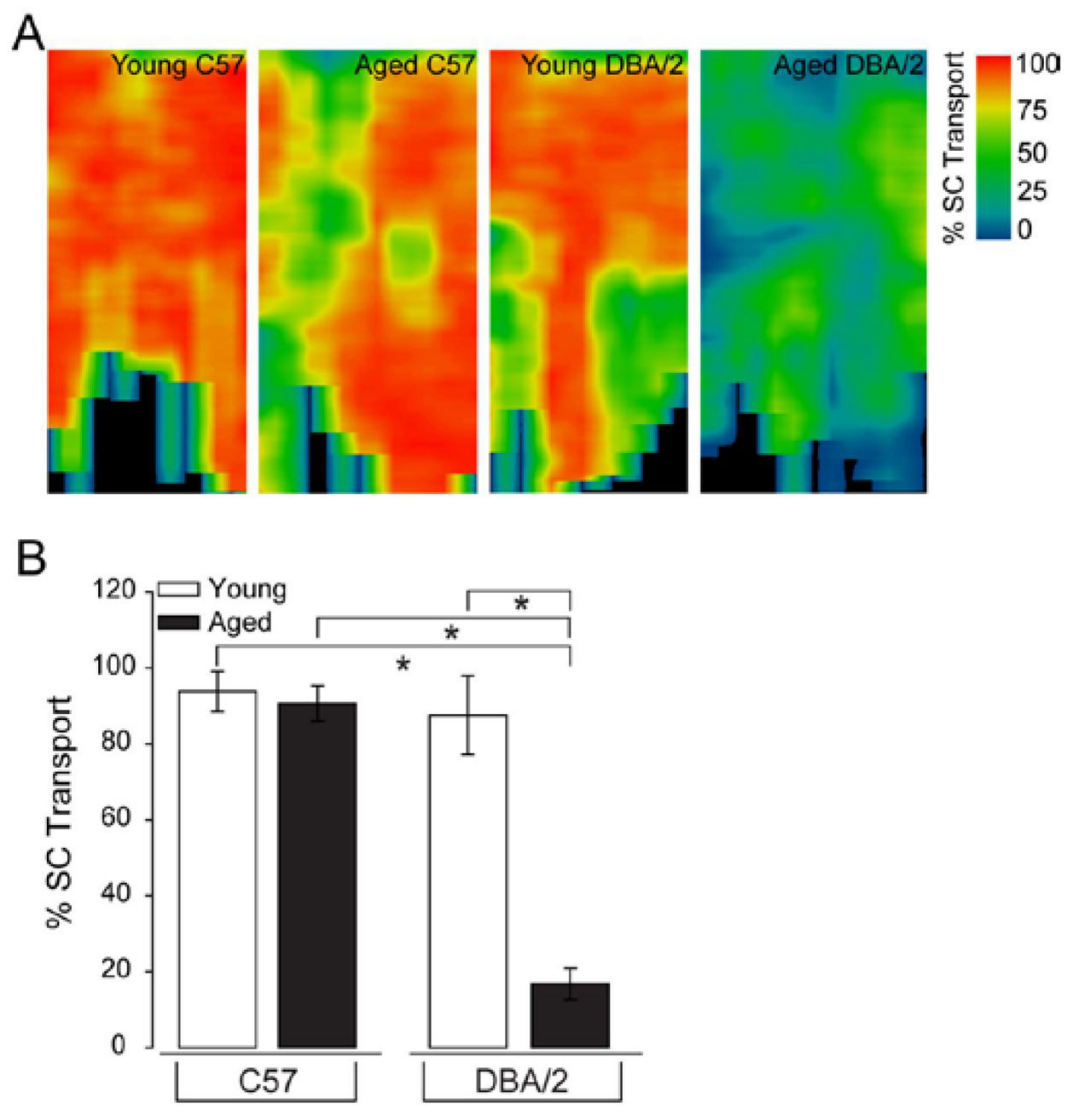
Glaucoma induces severe deficits in anterograde axonal transport by RGCs. (A) Representative, two-dimensional spatial maps of CTB labeling across entire retinotopic region of one lobe of the superior colliculus from young C57, aged C57, young DBA/2 and aged DBA/2 mice. Color bar indicates percent of CTB transport to the superior colliculus (% SC Transport), where blue is 0% and red is 100%. Collicular maps from aged C57 and young DBA/2 mice reveal some sectorial decreases in CTB transport, but with a majority of the map demonstrating full transport. In contrast, aged DBA/2 retina exhibits drastic reduction in CTB transport that is also sectorial in nature. (B) Quantification of mean percent of CTB transport in the superior colliculus (% SC Transport; y-axis) reveals that CTB transport to the colliculus is severely compromised in aged DBA/2 mice, as compared to young C57, aged C57 and young DBA/2 mice. Error bars represent ± standard deviation and asterisks indicate p<0.05.

**Figure 9:**
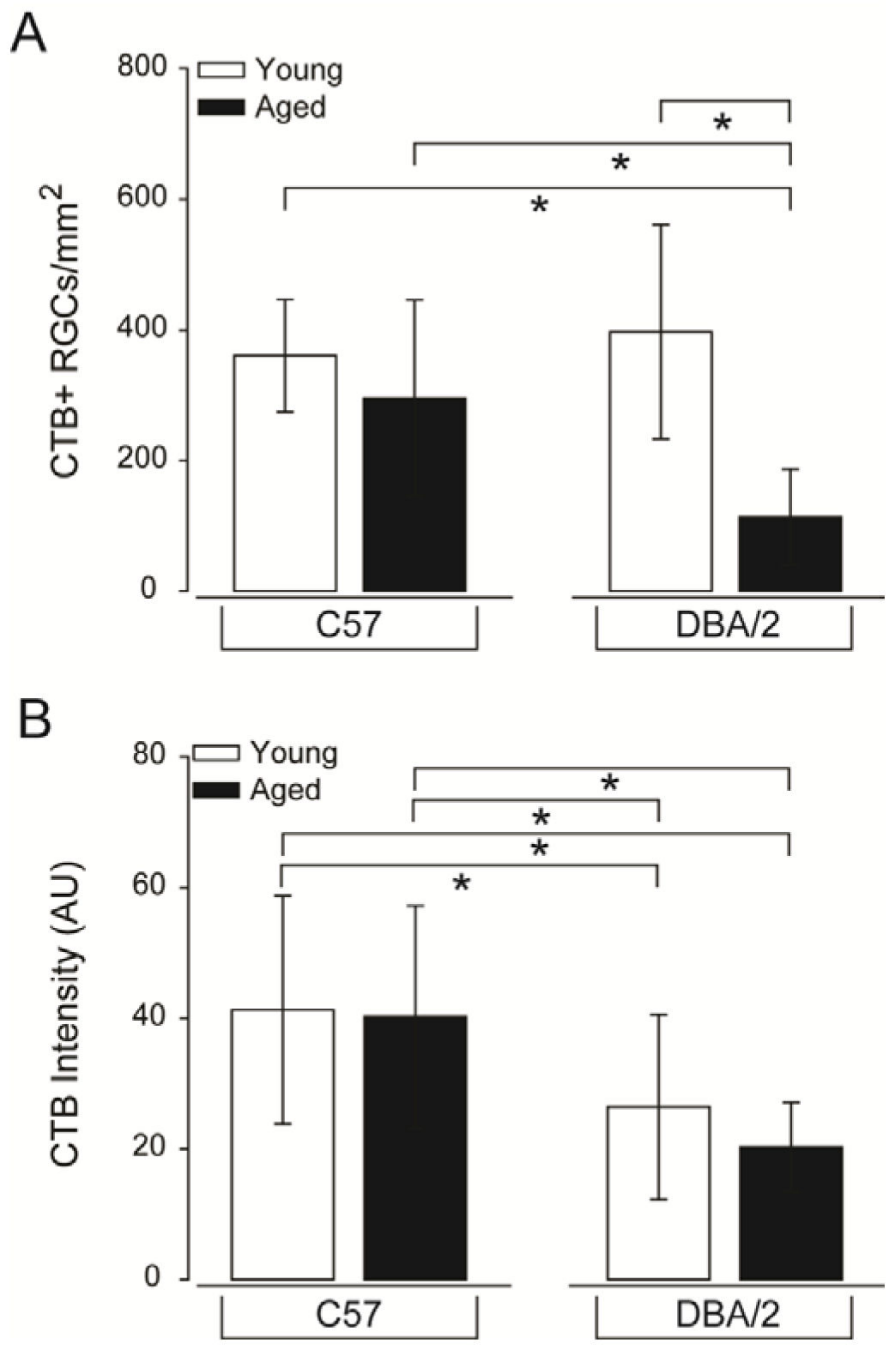
RGC health, as measured by active uptake of neural tracer, is severely compromised in glaucomatous retina. (A) Quantification of the density of RGCs with any level of CTB uptake above background levels represented as the density of CTB+ RGCs/mm^2^. Aged DBA/2 retina exhibits a highly significant decrease in the density of RGCs with detectable uptake of the neural tracer CTB, as compared to young C57, aged C57 and young DBA/2 mice. Error bars represent ± standard deviation and asterisks indicate p<0.05. (B) Quantification of total CTB labeling intensity (arbitrary units; y-axis) reveals that while there is no significant reduction in the density of CTB+ RGCs in young DBA/2 retina, RGCs in both young DBA/2 and aged DBA/2 retina exhibit reduced uptake of CTB. Error bars represent ± standard deviation and asterisks indicate p<0.05.

In agreement with previous studies, we found significant deficits in anterograde transport of CTB from the retina to the superior colliculus of aged DBA/2 mice, as indicated by a primarily green-blue intensity map across the entire retinorecipient colliculus (Figure 8A) [3]. Young C57, aged C57 and young DBA/2 mice exhibited similar transport maps with markedly greater signal than aged DBA/2 mice, as indicated with primarily red-yellow intensity maps (Figure 8A). Quantification of CTB transport confirmed that there was no significant difference in axonal transport between young C57, aged C57 and young DBA/2 retina (p>0.05 for all; Figure 8B). In contrast, axonal transport was reduced by 79–82%, as compared to young C57, aged C57 and young DBA/2 mice (p<0.01 for all; Figure 8B). Similarly, analysis of CTB uptake in retina indicated an approximate 75% decrease in the density of CTB+ RGCs in aged DBA/2 retina compared to young C57, aged C57 and young DBA/2 retina (p<0.01 for all; Figure 9A). There was no significant difference in the density of CTB+ RGCs in young C57, aged C57 or young DBA/2 retina (p>0.05 for all; Figure 9A). Interestingly, both young and aged DBA/2 retina exhibited decreased total intensity of CTB labeling in retina, as compared to young C57 and aged C57 retina (Figure 9B). For young DBA/2 retina, total CTB intensity was 36% lower than young C57 and aged C57 retina (p<0.05; Figure 9B). For aged DBA/2 retina, total CTB intensity was 51% lower than young C57 and aged C57 retina (p<0.01; Figure 9B). There was no significant difference between young and aged C57 retina (p>0.05) or between young and aged DBA/2 retina (p>0.05; Figure 9B).

**Figure 10:**
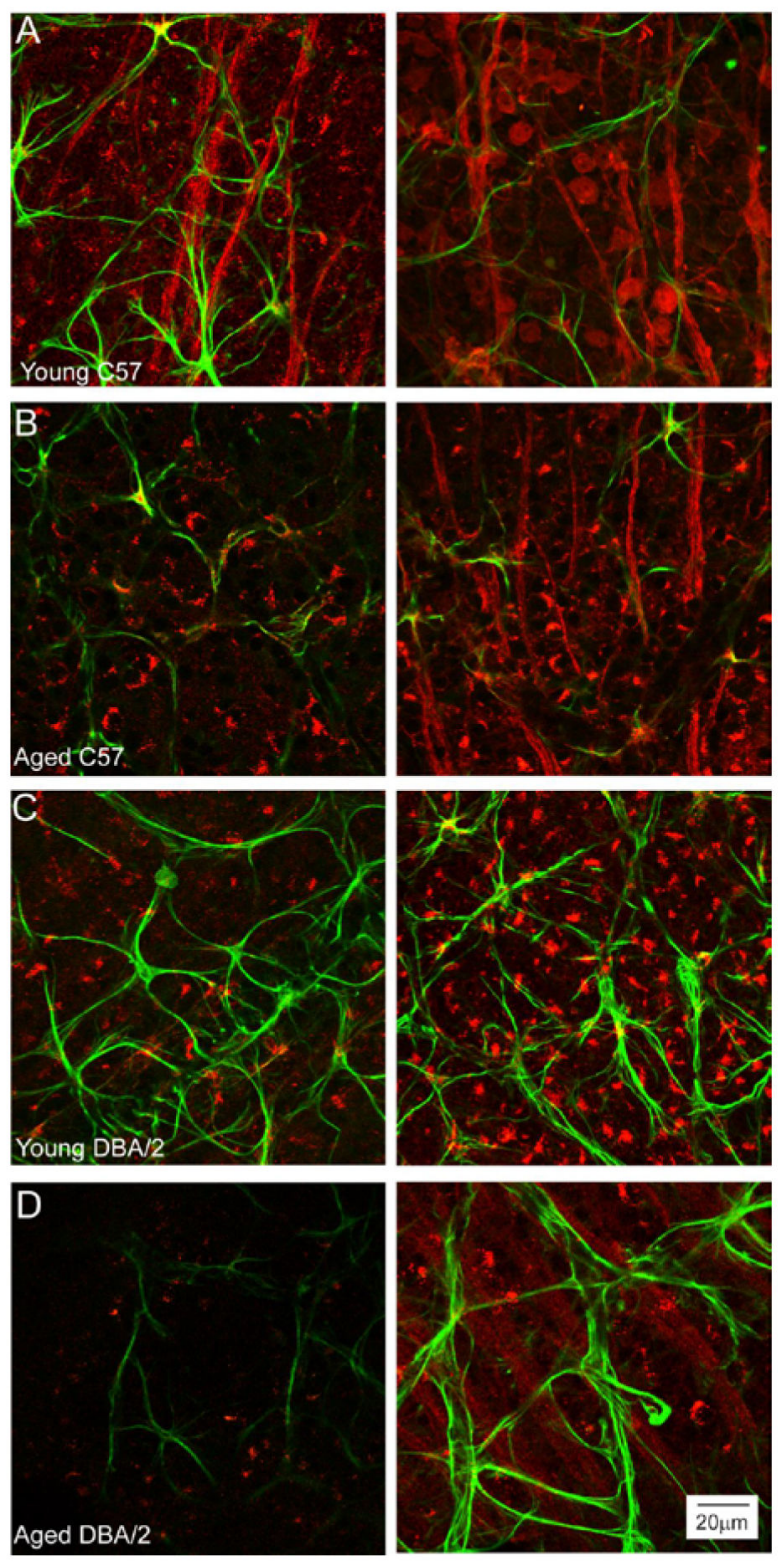
Spatial variability in RGC uptake of CTB coincides with morphological variations in spatially-coincident astrocytes. Representative confocal micrographs of wholemount retina from young C57 (A), aged C57 (B), young DBA/2 (C) and aged DBA/2 (D) mice with anterograde tracing of CTB (red) and immunolabeling against the astrocyte marker GFAP (green). Left and right columns illustrate the range of CTB labeling noted within individual retina, where the left column represents low intensity labeling and the right column represents high intensity labeling. Aged DBA/2 (D) retina exhibits the greatest degree of variability in CTB labeling intensity. However, aged C57 and young DBA/2 also exhibit some degree of spatial variability in CTB labeling. Astrocyte morphology appears to differ with respect to degree of CTB labeling aged C57, young DBA/2 and aged DBA/2 retina, particularly with regard to the area covered by GFAP+ astrocytes. Scale is the same for (A–D).

Qualitative assessment of spatially-coincident CTB labeling and GFAP immunoreactivity in retina revealed potential correlations between astrocyte morphology and the intensity of CTB label (Figure 10). As expected, young C57 retina exhibited rather consistent CTB labeling of RGCs and their axons across individual retina (Figure 10A). However, there were some variations in CTB localization within RGC soma, where it was concentrated primarily near the axon hillock in some soma (left panel; Figure 10A) and distributed evenly throughout the soma of others (right panel; Figure 10A). Qualitatively, it appeared that areas with more concentrated CTB exhibited a higher density of astrocytes (Figure 10A). Aged C57 retina exhibited more variability in CTB labeling than young C57 retina; however, CTB labeling was detectable in most RGC soma (Figure 10B). However, areas with lower levels of CTB appeared to have a greater density of astrocytes, compared to areas of high CTB labeling (right vs. left panels; Figure 10B). Similar to aged C57 retina, young DBA/2 retina exhibited some spatial variability in CTB labeling, but with all RGCs exhibiting some degree of CTB labeling (Figure 10C). As in aged C57 retina, areas of young DBA/2 retina with lower CTB labeling appeared to have a more complex network of astrocytes, if not greater density, than areas with higher CTB labeling (right vs. left panels; Figure 10C). In contrast, aged DBA/2 exhibited marked variations in CTB labeling that coincided with equally marked differences in morphology of spatially-coincident astrocytes, particularly with regard to astrocyte density (right vs. left panels; Figure 10D). Together, these data confirms that RGC health in glaucoma and, to a lesser degree, with exposure to glaucoma risk factors is spatially variable across individual retina and those astrocyte microdomains, as defined by reactivity indices, could potentially correlate with and perhaps serve as a biomarker for RGC health.

### Percent of retinal area covered by astrocytes is a viable index for glaucoma-related changes in astrocyte microdomains

**Figure 11:**
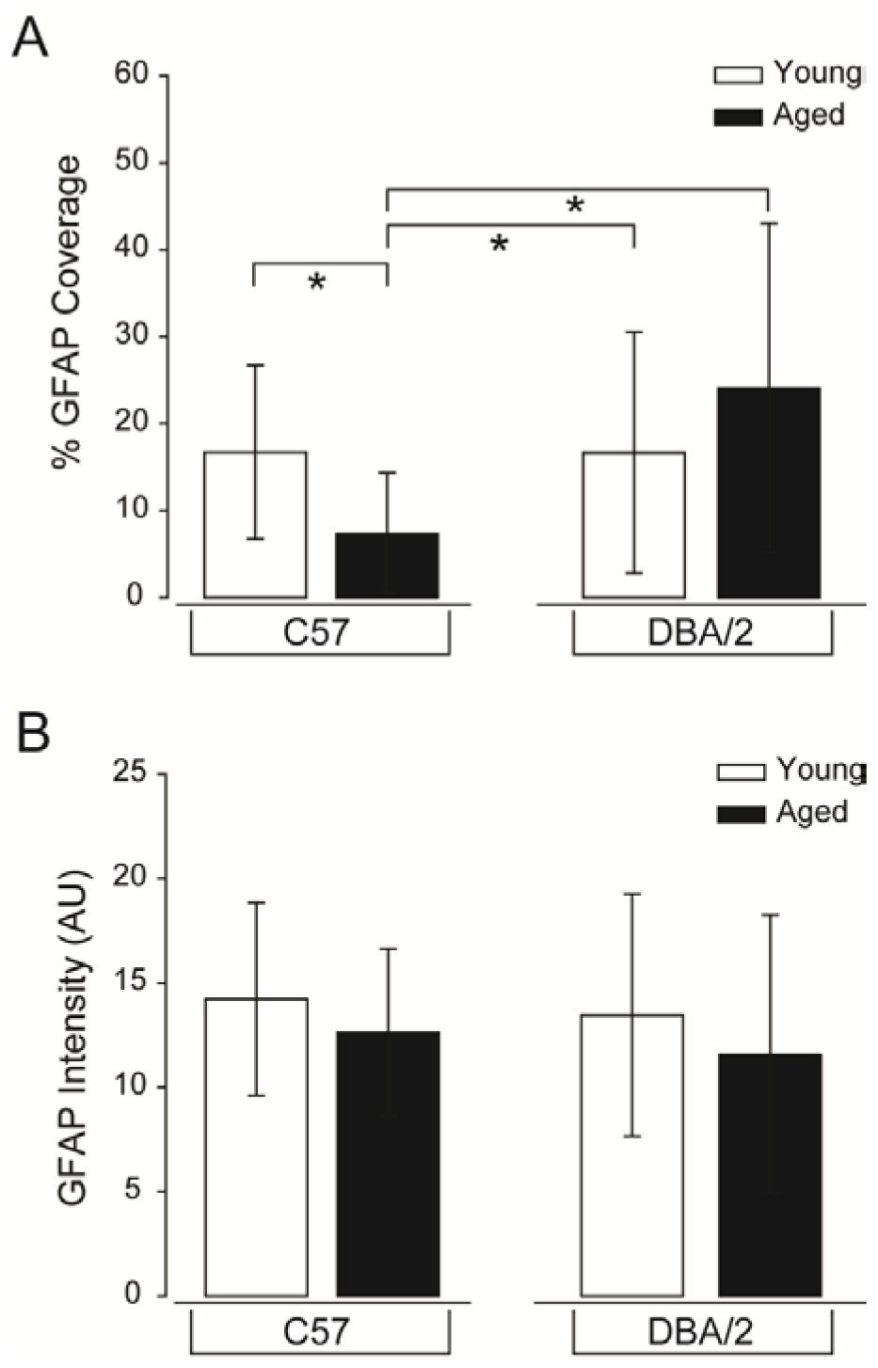
Percent of the retinal area covered by astrocytes is a viable index for glaucoma-induced changes in astrocyte microdomains. (A) Quantification of the percent of retinal area covered by astrocytes in 200 mm×200 mm microdomains, represented as % GFAP coverage (y-axis) reveals that normal aging induces a substantial decrease in astrocyte coverage, as compared to young C57 and young DBA/2 retina. In contrast, glaucomatous retina (aged DBA/2) demonstrates a greater than 3-fold increase in astrocyte coverage than its age-matched C57 control. Error bars represent ± standard deviation and asterisks indicate p<0.05. (B) Quantification of total GFAP labeling intensity (arbitrary units; y-axis) reveals that there is no significant difference in overall GFAP expression between conditions. Error bars represent ± standard deviation and asterisks indicate p<0.05.

To evaluate whether astrocyte microdomains could serve as a biomarker for RGC health, we first needed to develop an approach that could quickly and accurately identify astrocyte microdomains for potential translation to a clinical setting. Our data identify soma area and density as primary indicators of astrocyte microdomains for glaucomatous retina and retina with glaucoma risk factors. Both of these measures relate to the amount of retinal area covered by astrocytes. As such, we modified our astrocyte analysis to incorporate both indices into one index that addresses coverage area. To do so, we counted the number of pixels with any GFAP labeling, regardless of intensity, and divided this by the total number of pixels in each image. This provided a percentage value for coverage of retinal area by astrocytes. Since GFAP intensity was also relevant for defining astrocyte microdomains, we also measured for the total image. Using these calculations, we observed detectable differences in the percent of retinal area covered by astrocytes (% GFAP coverage) between healthy and disease-related states. As predicted by our hypertrophy and proliferation/migration analyses, aged C57 retina exhibited a 56% reduction in the mean retinal area covered by astrocytes, as compared to young C57 and young DBA/2 retina (p<0.01 for both; Figure 11A). Similarly, the mean retinal area covered by astrocytes was 3.5-fold greater in aged DBA/2 than aged-matched C57 retina, indicating a significant increase in astrocyte coverage with onset of glaucoma (p<0.01; Figure 11A). There was no significant difference in mean GFAP intensity (total) for any condition, which indicates that GFAP intensity is only relevant in the context on density and soma area (p>0.05 for all; Figure 11B). These data suggest that the percent of retinal area covered by astrocytes, which could be translated clinically, can detect glaucoma-related changes in astrocytes and that if spatially correlated with RGC health, could be a viable tool for delineating areas of retina at-risk for glaucoma-related vision loss.

### Percent of retinal area covered by astrocytes is a viable index for glaucoma-related changes in astrocyte microdomains

**Figure 12:**
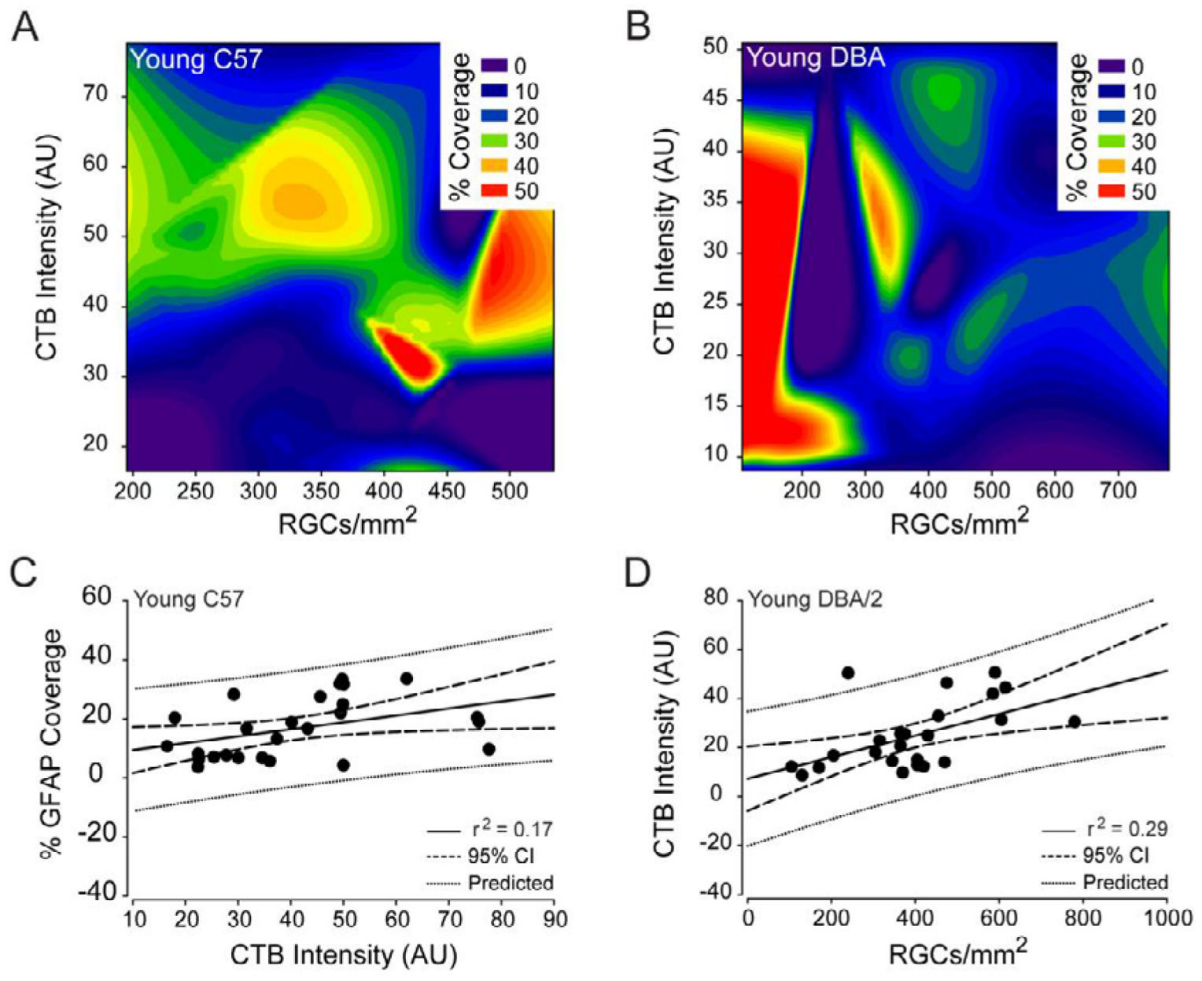
Percent coverage of retinal area by astrocytes is not a strong predictor of RGC health in young C57 or young DBA/2 retina. (A and B) Contour plot depicting the spatial relationships between percent coverage of retinal area by astrocytes (color bar; % coverage), CTB+ RGC density (x-axis; CTB+ RGCs/mm^2^) and total CTB intensity (y-axis; arbitrary units) in young C57 (A) and young DBA/2 (B) retina. (C) Regression graph of the positive correlation between total CTB intensity and percent coverage of retinal area by astrocytes in young C57 retina plotted as arbitrary units of intensity (x-axis) versus % GFAP coverage (y-axis). Solid lines indicate regression line based on r^2^ value. Dashed lines indicate the 95% confidence interval. Dotted lines indicate predicted values based on r^2^ and 95% confidence. (D) Regression graph of the positive correlation between CTB+ RGC density and total CTB intensity in young DBA/2 retina plotted as CTB+ RGCs/mm^2^ (x-axis) versus arbitrary units of intensity (y-axis). Solid lines indicate regression line based on r^2^ value. Dashed lines indicate the 95% confidence interval. Dotted lines indicate predicted values based on r^2^ and 95% confidence.

To determine whether percent of retinal area covered by astrocytes is a biomarker for RGC health in retina, we performed correlation and regression analysis of astrocyte coverage area (% GFAP coverage), total GFAP intensity, total CTB intensity and CTB+ RGC density in spatially-coincident samples. In young C57 retina, % GFAP coverage positively correlated with total CTB intensity (coeff=0.41, p=0.03; Figure 12A). This was the only significant correlation between astrocyte and RGC health indices for healthy retina (p>0.05 for all; Figure 12A). In contrast, young DBA/2 retina exhibited no significant correlations between astrocyte and RGC health indices (p>0.05 for all; Figure 12B). However, there was a trend towards a negative correlation between % GFAP coverage and the density of CTB+ RGCs (coeff=−0.37, p=0.06; Figure 12B). For RGC-related indices alone, there was a positive correlate between total CTB intensity and the density of CTB+ RGCs (coeff=0.54, p<0.01; Figure 12B). Regression analysis revealed that the tendency for the percent of retinal area covered by astrocytes to increase with increasing CTB label in young C57 retina had a predictive capacity of 17% (r^2^=0.17, p=0.03; Figure 12C). The positive correlation between total CTB intensity and the density of CTB+ RGCs in young DBA/2 retina was more reliable with a predictive capacity of 28% (r^2^=0.28, p<0.01; Figure 12D).

**Figure 13:**
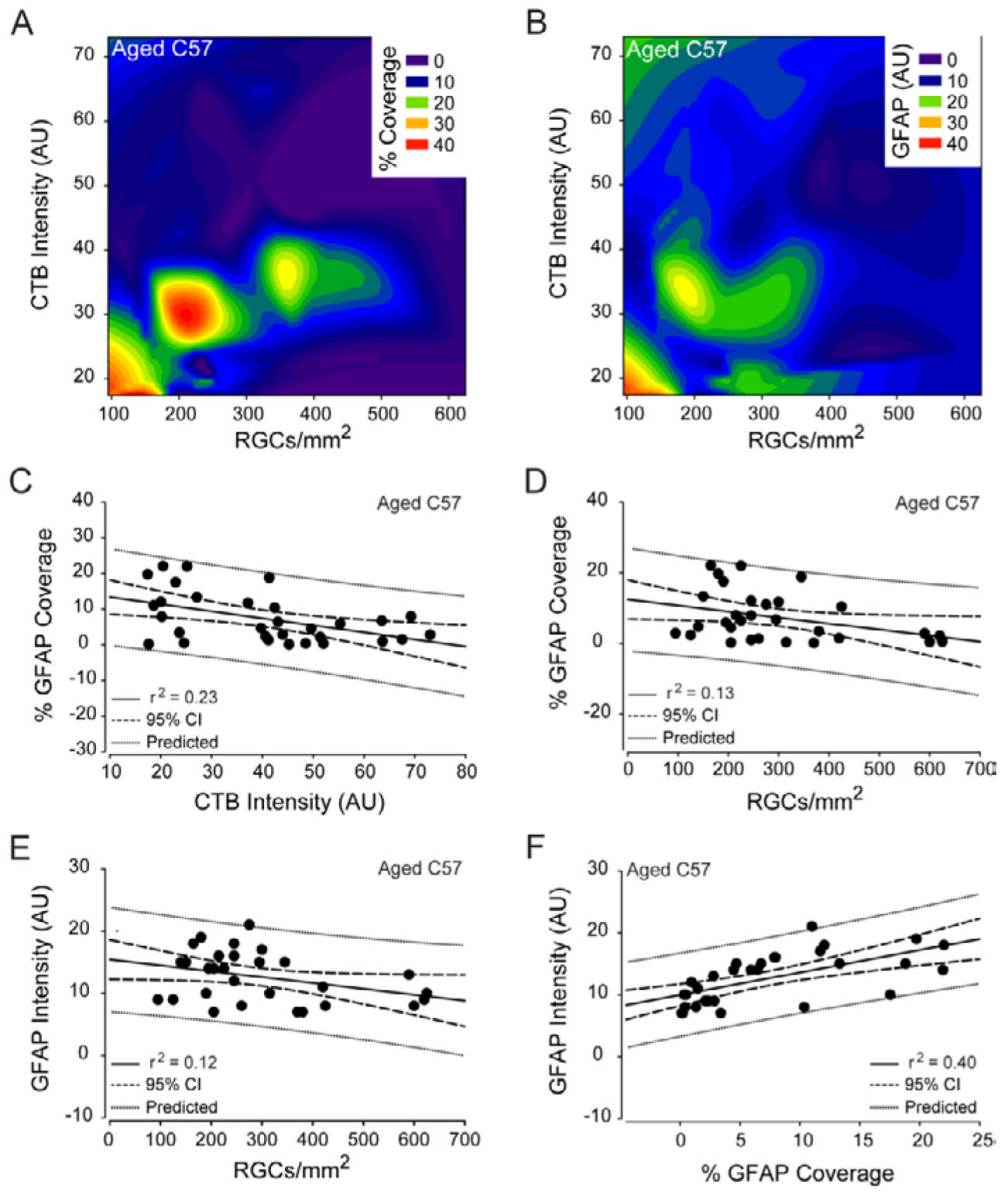
Astrocyte reactivity predicts RGC health in normal aging. (A) Contour plot depicting the spatial relationships between percent coverage of retinal area by astrocytes (color bar; % coverage), CTB+ RGC density (x-axis; CTB+ RGCs/mm^2^) and total CTB intensity (y-axis; arbitrary units) in aged C57 retina. (B) Contour plot depicting the spatial relationships between total GFAP intensity (color bar; GFAP (AU)), CTB+ RGC density (x-axis; CTB+ RGCs/mm^2^) and total CTB intensity (y-axis; arbitrary units) in aged C57 retina. (C) Regression graph of the negative correlation between total CTB intensity and percent coverage of retinal area by astrocytes in aged C57 retina plotted as arbitrary units of intensity (x-axis) versus % GFAP coverage (y-axis). (D) Regression graph of the negative correlation between CTB+ RGC density and percent coverage of retinal area by astrocytes in aged C57 retina plotted as CTB+ RGCs/mm^2^ (x-axis) versus % GFAP coverage (y-axis). (E) Regression graph of the negative correlation between CTB+ RGC density and total GFAP intensity in aged C57 retina plotted as CTB+ RGCs/mm^2^ (x-axis) versus arbitrary units of GFAP intensity (y-axis). (F) Regression graph of the positive correlation between percent coverage of retinal area by astrocytes and total GFAP intensity in aged C57 retina plotted as % GFAP coverage (x-axis) versus arbitrary units of GFAP intensity (y-axis). For all graphs, solid lines indicate regression line based on r^2^ value. Dashed lines indicate the 95% confidence interval. Dotted lines indicate predicted values based on r^2^ and 95% confidence.

In contrast to young C57 and DBA/2 retina, aged C57 retina exhibited the greatest number of correlative relationships between astrocyte reactivity and RGC health indices. In normal aging, the percent retinal area covered by astrocytes was negatively correlated with both the total intensity of CTB label (coeff=−0.48, p<0.01) and the density of CTB+ RGCs (coeff=−0.37, p=0.04; Figure 13A). Similarly, total GFAP intensity also negatively correlated with the density of CTB+ RGCs (coeff=−0.35, p=0.05; Figure 13B). Finally, both indices of astrocyte reactivity were positively correlated (coeff=0.63, p<0.01; Figures 13A and 13B). The percent retinal area covered by astrocytes was almost 2 times better at predicting CTB label (r^2^=0.23, p<0.01) than density of CTB+ RGCs (r^2^=0.13, p=0.05), as indicated by predictive capacities of 23% and 13%, respectively (Figures 13C and 13D). The ability for total intensity of GFAP to predict the density of CTB+ RGCs was similar to that of percent area coverage, with a predictive capacity of 12% (r^2^=0.12, p=0.05; Figure 13E). Since both percent area coverage and total GFAP intensity were capable of predicting the density of CTB+ RGCs, it is not surprising that these two indices of astrocyte reactivity had a high predictive capacity of 40% (r^2^=0.40, p<0.01; Figure 13F).

**Figure 14:**
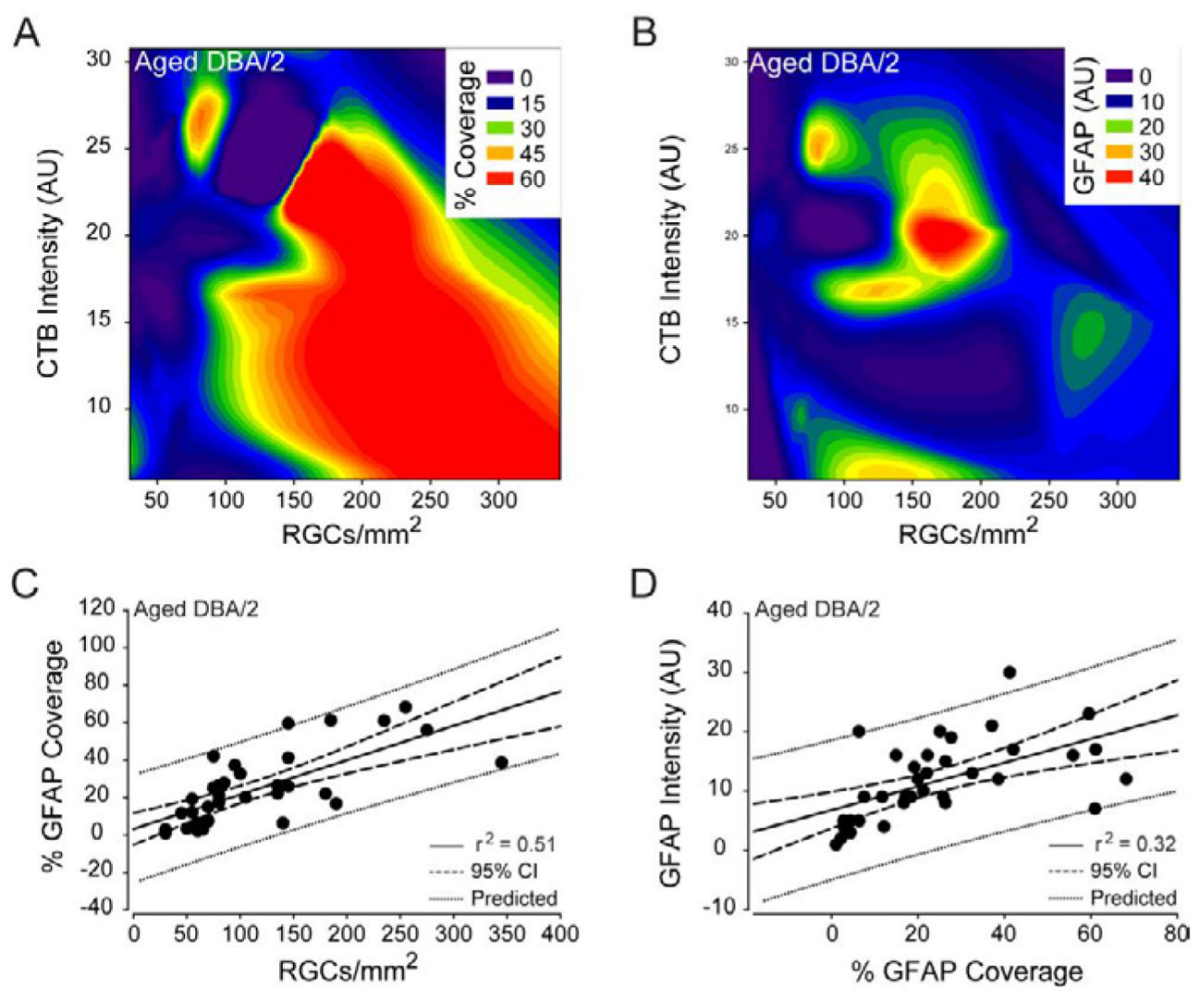
Percent coverage of retinal area by astrocytes is a strong predictor of RGC health in glaucomatous retina. (A) Contour plot depicting the spatial relationships between percent coverage of retinal area by astrocytes (color bar; % coverage), CTB+ RGC density (x-axis; CTB+ RGCs/mm^2^) and total CTB intensity (y-axis; arbitrary units) in aged DBA/2 retina. (B) Contour plot depicting the spatial relationships between total GFAP intensity (color bar; GFAP (AU)), CTB+ RGC density (x-axis; CTB+ RGCs/mm^2^) and total CTB intensity (y-axis; arbitrary units) in aged DBA/2 retina. (C) Regression graph of the positive correlation between CTB+ RGC density and percent coverage of retinal area by astrocytes in aged DBA/2 retina plotted as CTB+ RGCs/mm^2^ (x-axis) versus % GFAP coverage (y-axis). Solid lines indicate regression line based on r^2^ value. Dashed lines indicate the 95% confidence interval. Dotted lines indicate predicted values based on r^2^ and 95% confidence. (D) Solid lines indicate regression line based on r^2^ value. Dashed lines indicate the 95% confidence interval. Dotted lines indicate predicted values based on r^2^ and 95% confidence. Regression graph of the positive correlation between percent coverage of retinal area by astrocytes and total GFAP intensity in aged DBA/2 retina plotted as % GFAP coverage (x-axis) versus arbitrary units of GFAP intensity (y-axis).

While aged C57 exhibited the largest number of correlative and predictive relationships between astrocyte reactivity and RGC health, aged DBA/2 exhibited the most reliable relationship. For aged DBA/2 retina, the only statistically significant relationship between astrocyte reactivity and RGC health was a positive correlation between % area coverage and the density of CTB+ RGCs (coeff=0.71, p<0.01; Figure 14A). It is important to note that this relationship was opposite of that observed in age-matched C57 retina (Figure 13A). Aged DBA/2 retina also exhibited a trend towards a positive correlation between total GFAP intensity and density of CTB+ RGCs that was not quite statistically significant (coeff.=0.32, p=0.06; Figure 14B). Like aged C57 retina, % area covered and total GFAP intensity were positively correlated in aged DBA/2 retina (coeff=0.56; p<0.01; Figure 14B). The tendency for retinal areas with low density of CTB+ RGCs to also have less astrocyte coverage had a predictive capacity of 51% (r^2^=0.51, p<0.01; Figure 14C). The tendency for astrocytes in areas with greater astrocyte coverage to express higher levels of GFAP had a similar predictive capacity of 32% (r^2^=0.28, p<0.01), which was similar to the same relationship in aged C57 retina (Figure 14D). Together, these data suggest that the percent of retinal area covered by astrocytes is a substantial predictor of RGC health in glaucomatous retina. Interestingly, astrocyte reactivity can also predict, although with less accuracy, RGC health in aging retina, the primary risk factor for glaucoma. This stands in sharp contrast to healthy retina and retina genetically predisposed to glaucoma, for which astrocyte reactivity is generally a poor indicator of RGC health.

## Discussion

In glaucoma, a disease characterized by progressive loss of RGCs and their axons, astrocyte reactivity is well-established as a key player in pathophysiology of the retina, optic nerve and visual centers of the brain [7,11–24]. That neurodegeneration of RGCs in glaucoma progresses in a sectorial pattern that is preceded by deficits in axonal transport suggests that there is an earlier window for therapeutic treatment than is currently being utilized. The neuroinflammatory events that accompany RGC degeneration are also likely to be spatially-regulated and may provide an avenue for alternative treatment and diagnostics. Here we examined the spatial characteristics of astrocyte reactivity (migration/proliferation, hypertrophy and GFAP expression) in healthy retina, retina with two glaucoma-related risk factors (aging and genetic predisposition) and glaucomatous retina and established relationships between these reactivity indices and the spatial organization of astrocytes as well as RGC health. Our findings suggest that microdomains of astrocyte reactivity are biomarkers for functional decline of RGCs, which may have potential for clinical application. The implications of our findings are discussed in detail below.

Our analyses of individual indices of astrocyte reactivity revealed several key characteristics of astrocyte reactivity in glaucomatous retina and retina with glaucoma risk factors. First, the mean density of astrocytes did not change in response to glaucoma risk factors or glaucoma itself. Instead, our histogram analyses revealed that astrocytes are re-distributed to produce areas of lower or higher astrocyte density. Interestingly, severity of the stressor determines the degree of variability in astrocyte density throughout regions of the retina, with glaucomatous retina exhibiting the greatest variation. Second, retina with risk factors for glaucoma (aged C57 and young DBA/2) exhibited a tendency towards shrinkage of astrocytes (hypotrophy), suggesting that astrocyte hypotrophy may be of interest in examining the pathophysiology of glaucoma risk and onset. Finally, the mean GFAP expression per cell was greatest in healthy retina, which exhibited the least spatial variability across all astrocyte reactivity indices. Histogram analysis revealed that while some groups of astrocytes exhibited high levels of GFAP expression in retina challenged by glaucoma-related stressors, a majority of the astrocyte population had decreased levels of GFAP expression. In combination with our findings regarding astrocyte hypotrophy in response glaucoma risk factors, these data may indicate that astrocyte decline could be a relevant factor glaucoma onset and progression. This is supported by recent evidence in optic nerve and elsewhere in the central nervous system, suggesting that astrocyte decline may contribute to neurodegeneration [33,34].

To determine whether spatial variations in indices of astrocyte reactivity were inter-related, we performed correlation and regression analysis. We found that individual indices of astrocyte reactivity could reliably predict one another in spatially-coincident astrocytes. This indicates that astrocytes in close proximity share morphological characteristics relevant to their reactivity states, which suggests the formation of astrocyte microdomains that not only underlie increased variability noted in our morphological analyses, but also likely have distinct functional significance. These findings are in accordance with recent work describing spatial variations in both microglia reactivity and IL-6 signaling in DBA/2 retina [15,17].

Although the degree of predictability between reactivity indices was stressor-dependent, the most striking finding was that measures of density and hypertrophy were most consistent in their ability to predict astrocyte organization. That both density and hypertrophy are related to the amount of retinal area covered by astrocytes suggests that the degree of contact between astrocytes and the surrounding neural and vascular structures is a key defining microdomains of reactivity. When using percent of retina area covered by astrocytes as an index of astrocyte microdomains, we found a dramatic decrease in astrocyte coverage in aged C57 retina, which again suggests that astrocyte decline may be contribute to risk for neurodegeneration. We found that in aged DBA/2 mice the area of astrocyte coverage increased more than 3-fold from coverage quantified in age-matched C57 mice, which suggests a link between elevated IOP and area of astrocyte coverage. Our results in glaucomatous retina are contrary to previously published studies of astrocyte coverage in the optic nerve of aged DBA/2 mice [23] and in retina following acute IOP elevation [14], which both demonstrate a reduction in astrocyte coverage. With regard to the study by Ramírez et al. [14], this discrepancy is likely attributable to the chronic nature of our glaucoma model (12 months) versus the acute nature of their model (3 weeks). Although we used the same model as Lye-Barthel et al. [23], they described area of astrocyte coverage in the optic nerve, where astrocytes are directly impacted by IOP-induced mechanical strain and exhibit both constitutive and pathological responses that differ from those in the retina [11,13,16,18,20–22].

A hallmark of the slow, progressive degeneration of RGCs in glaucoma is failure in both anterograde and retrograde axonal transport [1–8]. These functional deficits precede structural loss of RGCs and their axons and are accompanied by a variety of pathological events, including glial cell reactivity and cytokine signaling [3,7,9,19]. These studies suggest that there is a larger therapeutic window for glaucoma treatments that begins prior to loss of RGCs and subsequent visual deficits. Unfortunately, translating detection of this earlier therapeutic window into a diagnostic measure for the clinic has proven difficult [35]. Here we found that percent retinal area covered by astrocytes could reliably predict the exact density of CTB+ RGCs with an accuracy of greater than 50%. In a clinical setting, calculation of exact RGC density is unnecessary; therefore, the potential for this index to detect regions of RGCs with failing axonal transport may be much higher when it is considered in terms of estimating RGC health more broadly.

From a technical standpoint, visualization of astrocytes and measurement of astrocyte coverage area in the human retina is within the scope of current imaging technologies in the clinic [36,37]. In fact, a study in patients with primary open angle glaucoma suggests that activation of astrocytes and Müller glia, as assessed by time domain spectral coherence tomography, may actually lead to artifact when measuring retinal nerve fiber layer thickness [36]. Animal models also suggest that in vivo imaging of astrocytes can be facilitated by contrast dyes, including peripheral-type benzodiazepine receptor [38] and sulforhodamine 101 [39–42]. However, it is unclear, particularly for sulforhodamine 101, whether these dyes would reliably stain astrocytes in the nerve fiber layer of humans [43,44].

Together, these data suggest that: (1) astrocyte reactivity occurs in microdomains throughout glaucomatous retina as well as retina with risk factors for glaucoma, (2) these astrocyte microdomains are primarily differentiated by the degree of retinal area covered by the astrocytes within them and (3) percent retinal area covered by astrocytes has the potential to be a biomarker for decreased RGC health, which could allow practitioners to identify regions with faltering RGC health prior to overt nerve fiber layer or visual field defects.
